# Metabolomics based markers predict type 2 diabetes in a 14-year follow-up study

**DOI:** 10.1007/s11306-017-1239-2

**Published:** 2017-07-28

**Authors:** Jun Liu, Sabina Semiz, Sven J. van der Lee, Ashley van der Spek, Aswin Verhoeven, Jan B. van Klinken, Eric Sijbrands, Amy C. Harms, Thomas Hankemeier, Ko Willems van Dijk, Cornelia M. van Duijn, Ayşe Demirkan

**Affiliations:** 1 Department of Epidemiology, Erasmus Medical Center, Rotterdam, The Netherlands; 2 Faculty of Engineering and Natural Sciences, International University of Sarajevo, Sarajevo, Bosnia and Herzegovina; 3 Department of Biochemistry and Clinical Analysis, Faculty of Pharmacy, University of Sarajevo, Sarajevo, Bosnia and Herzegovina; 4 Center for Proteomics and Metabolomics, Leiden University Medical Center, Leiden, The Netherlands; 5 Department of Internal Medicine, Section Pharmacology Vascular and Metabolic diseases, Erasmus Medical Center, Rotterdam, The Netherlands; 6 Division of Analytical Biosciences, Leiden Academic Centre for Drug Research, Leiden University, Leiden, The Netherlands; 7 Netherlands Metabolomics Centre, Leiden University, Leiden, The Netherlands; 8 Department of Human Genetics, Leiden University Medical Center, Leiden, The Netherlands; 9 Department of Endocrinology, Leiden University Medical Center, Leiden, The Netherlands

**Keywords:** Type 2 diabetes, Prediction, Metabolomics, Early biomarkers, Metabolites, Prospective study

## Abstract

**Background:**

The growing field of metabolomics has opened up new opportunities for prediction of type 2 diabetes (T2D) going beyond the classical biochemistry assays.

**Objectives:**

We aimed to identify markers from different pathways which represent early metabolic changes and test their predictive performance for T2D, as compared to the performance of traditional risk factors (TRF).

**Methods:**

We analyzed 2776 participants from the Erasmus Rucphen Family study from which 1571 disease free individuals were followed up to 14-years. The targeted metabolomics measurements at baseline were performed by three different platforms using either nuclear magnetic resonance spectroscopy or mass spectrometry. We selected 24 T2D markers by using Least Absolute Shrinkage and Selection operator (LASSO) regression and tested their association to incidence of disease during follow-up.

**Results:**

The 24 markers i.e. high-density, low-density and very low-density lipoprotein sub-fractions, certain triglycerides, amino acids, and small intermediate compounds predicted future T2D with an area under the curve (AUC) of 0.81. The performance of the metabolic markers compared to glucose was significantly higher among the young (age < 50 years) (0.86 vs. 0.77, p-value <0.0001), the female (0.88 vs. 0.84, p-value =0.009), and the lean (BMI < 25 kg/m^2^) (0.85 vs. 0.80, p-value =0.003). The full model with fasting glucose, TRFs, and metabolic markers yielded the best prediction model (AUC = 0.89).

**Conclusions:**

Our novel prediction model increases the long-term prediction performance in combination with classical measurements, brings a higher resolution over the complexity of the lipoprotein component, increasing the specificity for individuals in the low risk group.

**Electronic supplementary material:**

The online version of this article (doi:10.1007/s11306-017-1239-2) contains supplementary material, which is available to authorized users.

## Introduction

Early lifestyle intervention is a cost-effective recommendation to reduce the incidence of type 2 diabetes (Knowler et al. 2002; Li et al. 2010; Nanditha et al. 2014), asking for informative, sensitive and specific markers. Although the standard laboratory tests, such as fasting glucose, 2-h postprandial glucose, and glycated hemoglobin A1c (HbA1c), provide strong evidence for the risk of type 2 diabetes(Haffner et al. 1990; Shaw et al. 1999; Droumaguet et al. 2006), these predictors emerge after years of subclinical metabolic dysfunction (Tabak et al. 2009). Traditional risk factors (TRFs) such as age, sex, body mass index (BMI), and waist circumference also explain considerable part of future risk (Gray et al. 2010; Wilson et al. 2007), but fail to capture the full complexity of the etiology and their predictive performance vary between different risk groups (Kengne et al. 2014). BMI has been put forward as the modifiable risk factor but, there are also metabolically unhealthy normal weight (MUHNW) as well as metabolically healthy obese (MHO) individuals, raising the question to what extent BMI explain the mechanisms of the underlying metabolic disease (Mathew et al. 2016). Therefore, there is an increasing interest in finding informative markers that indicate the particular metabolic dysfunctions before the manifestation of the disease. Hence, people identified at high risk would be able to take preventive lifestyle interventions or treatments targeted to their individual molecular profile, eventually personalizing their health care.

High throughput metabolomics offers an opportunity to test multiple metabolic markers in large settings. Such approach led to the discovery of five amino acids by the prospective Framingham Heart Study (FHS) using a 12-year follow-up (Wang et al. 2011). Branched chain amino acids (BCAA) from this panel were previously pointed out in a case-control setting (Suhre et al. 2010) and later in a follow-up study of limited size (Lu et al. 2016; Yu et al. 2016). Other metabolites including phospholipids, triglycerides, acyl-carnitines, organic acids and small molecular weight compounds were also added to the list of metabolomics based predictors (Floegel et al. 2013; Walford et al. 2014; Wang-Sattler et al. 2012; Lu et al. 2016; Yu et al. 2016; Suhre et al. 2010), covering the glucose and phospholipid metabolism. However, lipoprotein metabolism, which is one of the key components of metabolic dysfunction, has not been addressed.

In the present study, we aimed to identify novel metabolic markers using a total of 261 metabolic features measured by either targeted mass spectrometry (MS) or by targeted nuclear magnetic resonance (NMR). The chemical classes of tested molecules include sub-fractions of lipoproteins, triglycerides, phospholipids, amino acids, and small intermediate compounds. We estimated the predictive performance of the selected marker set in comparison to other well-known predictors, including fasting glucose, TRFs, and the validated panel of amino acids.

## Research design and methods

### Study population

The Erasmus Rucphen Family genetic isolate study (ERF) is a prospective family based study located in Southwest of the Netherlands. This young genetic isolate was founded in the mid-eighteenth century and minimal immigration and marriages occurred between surrounding settlements due to social and religious reasons. The ERF study population includes 3465 individuals that are living descendants of 22 couples with at least six children baptized. Informed consent has been obtained from patients where appropriate. The study protocol was approved by the medical ethics board of the Erasmus Medical Center Rotterdam, the Netherlands (Santos et al. 2006).

The baseline demographic data and measurements of the ERF participants were collected around 2002–2006. All the participants filled out questionnaires on socio-demographics, diseases and medical history and lifestyle factors, and were invited to the research center for an interview and blood collection for biochemistry and physical examinations including blood pressure and anthropometric measurements have been performed. The participants were asked to bring all their current medications for registration during the interview. Venous blood samples were collected after at least 8 h fasting. Hypertension was defined as systolic blood pressure ≥140 mmHg or diastolic blood pressure ≥90 mmHg or treatment for hypertension. The family history was coded as 0, 1, 2 based on no first-degree relatives has type 2 diabetes, one has type 2 diabetes and more than one have type 2 diabetes. Baseline type 2 diabetes was defined according to the fasting plasma glucose ≥7.0 mmol/L and/or anti-diabetic treatment, yielding 212 cases and 2564 controls, totaling up to 2776. The follow-up data collection of the ERF study took place from March 2015 to May 2016 (9–14 years after baseline visit). During the follow up a total of 1935 participants’ records were scanned for incidence of type 2 diabetes in general practitioner’s databases. Additionally, a questionnaire on type 2 diabetes medication surveyed on 1232 participants in June 2010 (4–8 years after baseline visit) was referred if a participant were not included in May 2016 follow-up. This effort yielded the inclusion of 18 otherwise missed extra cases. To summarize, out of the 2564 controls at baseline, 1571 were followed-up for a mean 11.3 years (inter quartile range 11.0–12.2). Among those, 137 developed type 2 diabetes, whereas 1434 did not, comprising together the analytical sample for prediction analysis.

### Metabolomics measurements

In total 261 metabolic marker molecules including sub-fractions of lipoproteins, triglycerides, phospholipids, amino acids and small intermediate compounds, which throughout this article will be referred as “*metabolites*”, were measured by three different targeted platforms, either by NMR spectrometry or MS at baseline. The samples included in metabolomics measurements were not selected based on any disease. The platforms used in this research are: (1) Liquid Chromatography-MS (LC-MS, 116 positively charged lipids, comprising of 39 triglycerides (TG), 47 phosphatidylcholines (PC), 8 phosphatidylethanolamines (PE), 20 sphingolipids (SM), and 2 ceramides (Cer), available in up to 2638 participants) measured in Netherlands Metabolomics Center, Leiden using the method described before (Gonzalez-Covarrubias et al. 2013), (2) small molecular compounds window based NMR spectroscopy (NMR-COMP, 41 molecules comprising of 29 low-molecular weight molecules and 12 amino acids available in up to 2639 participants) measured in Center for Proteomics and Metabolomics, Leiden University Medical Center (Demirkan et al. 2015; Verhoeven et al. 2017), (3) lipoprotein window based NMR spectroscopy (NMR-LIPO, 104 lipoprotein particles size sub-fractions comprising of 28 very low-density lipoprotein (VLDL) components, 30 high-density lipoprotein (HDL) components, 35 low-density lipoprotein (LDL) components, 5 IDL components and 6 plasma totals, available in up to 2609 participants) measured in Proteomics and Metabolomics, Leiden University Medical Center and lipoprotein sub-fraction concentrations were determined by the Bruker algorithm (Bruker BioSpin GmbH, Germany) details were given previously (Kettunen et al. 2016). Details over the quality control of samples in these platforms can be found in the Supplementary Information. The laboratories had no access to phenotype information and the data pre-filtering and quality control for measurement errors were based on internal controls and duplicates.

### Metabolite identification

The compounds measured by LC-MS and NMR-COMP were identified according to the metabolomics standards initiative (MSI) level 1 using information coming from at least 2 different sources (Sansone et al. 2007). The available ChEBI ID were shown in Supplementary Table 1.

For metabolites measured by LC-MS, the identities of the lipids were assigned on the basis of accurate mass, fragmentation pattern, and retention times matched to authentic standards where available. The detail of the metabolite identification can be found in previous publications (Hu et al. 2008).

For metabolites measured by NMR-COMP, the identities of the small components and molecules were assigned by the peaks which are annotated using the combined information from chemical shift databases, spiking experiments, and correlation behaviors. The detail of the metabolite identification can be found in the methodological paper (Verhoeven et al. 2017).

For lipoproteins measured by NMR-LIPO, the method is based on the analysis of signals in the ^1^H-NMR spectrum which are related to the lipoproteins. Differences in lipoprotein composition, size and density translate into respective signal line shape differences, which can be used to extract information on lipoprotein main- and subclasses. As these are not real metabolites, the MSI criteria do not apply.

### Statistical methods

**Fig. 1:**
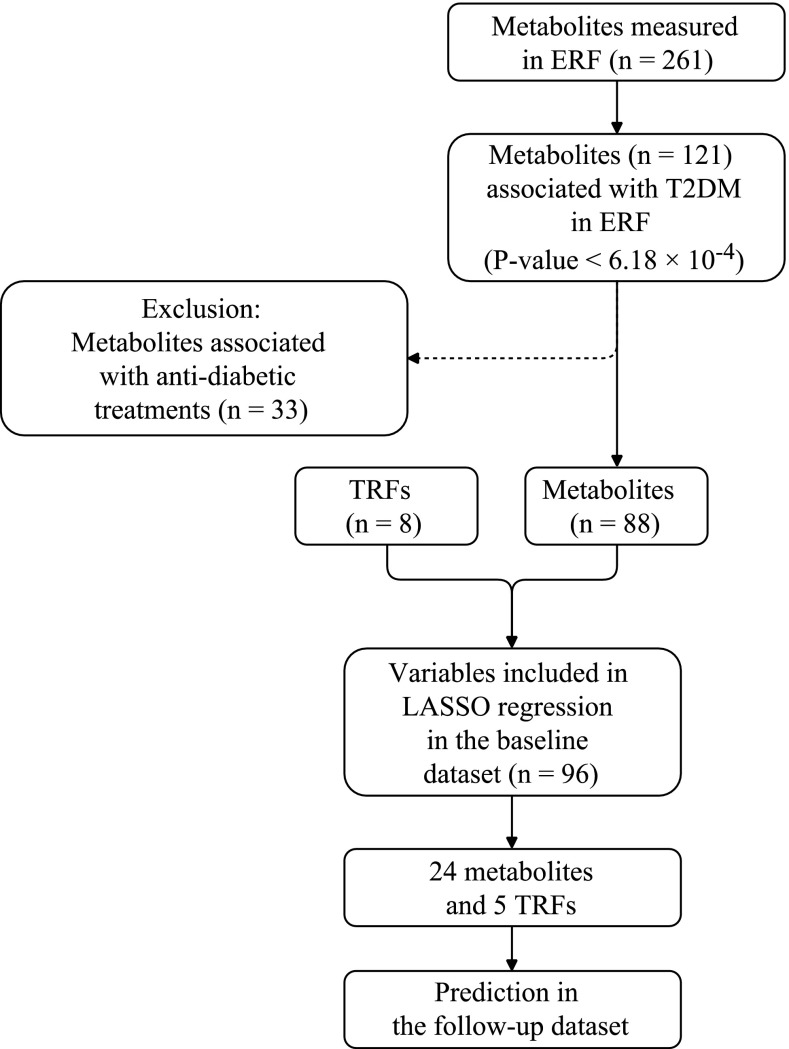
Flow chart of the metabolite selection

The distributions of individual metabolites were checked for non-normality by eye and outlying values that were more than four times standard deviation away from the mean were excluded from analysis. Non-normally distributed measurements were natural logarithm transformed, or rank transformed. Figure 1 shows the procedure that we followed for the selection of metabolites. Firstly, we tested the association between the 261 individual metabolites and prevalent type 2 diabetes using a logistic regression model adjusting for age, sex, and lipid-lowering medication. Residuals from the polygenic model (using “polygenic” function in the R package *GenABEL*), were used in all analysis to account for family relations among the ERF participants (Aulchenko et al. 2007). To control for multiple testing, we applied a Bonferroni correction based on the effect number of independent vectors in the data which were estimated to be 81 independent equivalents using Matrix Spectral Decomposition (MSD) (Li and Ji 2005). Thus, a p-value less than 6.18 × 10^− 4^ (0.05/81) was used as the threshold for metabolome-wide significance. We repeated analysis stratifying the cases into medicated and non-medicated cases to test if the associations were attributed to the effect of anti-diabetic medication. Metabolites that did not differentiate (p-value >0.05) between non-medicated diabetics (n = 68) versus controls (n = 2564) were not taken forward. These metabolite levels were assumed to be different due to the post medication metabolic changes in the diabetics. The remaining metabolites (n = 88, the list is given in Supplementary Table 1) and the TRFs (age, sex, family history, BMI, waist circumference, hypertension, HDL-cholesterol, and triglycerides) with scaled around 0 and standard deviation as 1 were included in the prior to LASSO (Least Absolute Shrinkage and Selection Operator) regression to select the set of predictors that maximize the prediction performance. The LASSO regression was performed using *glmnet* package in R (Friedman et al. 2010). We imputed these missing data points (i.e. 9.6–18.5% missing values) before selecting the independent predictors by LASSO regression which requires all the variables to be complete measurements. In order to select the best imputation method suitable for our data, we first generated a training dataset with 20% missing values at random and compared three methods: (1) deterministic imputation, (2) random regression imputation, (3) multiple imputation with R package “mice” (Andrew and Jennifer 2006). After comparing the results to the initial correlation with glucose and the means between the imputed values and real values for each method, multiple imputation was selected. The sum of predicted values from the multiple random regression model divided by the number of imputations (n = 20) was used to replace the missing data. The outliers more or less than four times standard deviation were removed after imputation. With the selected independent type 2 diabetes metabolic predictors, we assessed their associations with fasting glucose by linear regression analysis in the non-diabetic participants at baseline. To account for multiple testing in these 24 linear regression sets, a p-value <0.003 (0.05/16) was used as the threshold after MSD of the 24 metabolites that yielded 16 independent components.

### Prediction of incident type 2 diabetes

The metabolites selected from the baseline population were tested to predict the incidence of type 2 diabetes during the follow-up time. Area under the receiver operator characteristics (ROC) curve (AUC) of logistic regression together with continuous Net Reclassification Improvement (NRI) was performed to estimate the discrimination and reclassification in different models (Pencina et al. 2012). The models compared were: *ERF metabolite model* (the metabolites those selected in the current study only), *FHS metabolite model*, (the amino acids reported by the FHS research: isoleucine, leucine, valine, tyrosine, and phenylalanine), and the *TRF model* (age, sex, family history, BMI, waist circumference, hypertension, HDL-cholesterol, and triglycerides) and glucose only model (fasting plasma glucose measured at baseline) and combination of those. As some of the previous studies showed the association between metabolites and covariates, i.e. age, sex and BMI (Dunn et al. 2015; Lawton et al. 2008), we also tested the models in subgroups of sex, age (<50 vs. ≥50 years), and BMI (<25 vs. ≥25 kg/m^2^). A p-value <0.05 here was used as a cut off for significance improvement across the models. Meanwhile, the specificity with fixed 80% sensitivity in different prediction models is compared. Analyses were conducted using *R* (version 3.2.3).

## Results

Table 1 displays the baseline characteristics of the participants stratified by prevalent cases at baseline and incident cases in the follow-up. Compared to the participants who did not develop type 2 diabetes, those with type 2 diabetes were older, more often had a family history of the disease, suffered from hypertension, and have been using lipid-lowering medication. They had higher levels of BMI, waist circumference, blood pressure, triglycerides, fasting glucose, and lower levels of HDL-cholesterol. The participants with incident type 2 diabetes had higher fasting glucose at baseline compared to the individuals who did not develop type 2 diabetes during the follow up.

**Table 1:** Characteristics of the study population

	Baseline (n = 2776)		Follow-up (n = 1571)	
	Controls (n = 2564)	Cases (n = 212)	Controls (n = 1434)	Cases (n = 137)
Male [n (%)]	1132 (44.1)	108 (50.9)	595 (41.5)	78 (56.9)*
Age (years)	48.2 ± 14.3	59.8 ± 11.8*	47.7 ± 13.9	57 ± 10.7*
Diabetes in first-degree relatives				
0 individuals [n (%)]	1711 (76.6)	71 (55.0)	966 (76.4)	63 (53.8)
1 individual [n (%)]	428 (19.2)	37 (28.7)	248 (19.6)	38 (32.5)
≥2 individuals [n (%)]	95 (4.3)	21 (16.3)*	50 (4.0)	16 (13.7)*
Body mass index (kg/m^2^)	26.7 ± 4.6	30.0 ± 5.9*	26.6 ± 4.4	30.1 ± 5.1*
Waist circumference (cm)	86.7 ± 13.1	99.3 ± 14.2*	86.2 ± 12.8	98.9 ± 13.4*
Systolic blood pressure (mmHg)	139 ± 20	154 ± 21*	137.7 ± 19.6	152.4 ± 21.8*
Diastolic blood pressure (mmHg)	80.3 ± 10.0	82.9 ± 9.9	79.7 ± 9.6	84.8 ± 9.8*
Hypertension [n (%)]	1282 (50)	170 (80.2)*	674 (47.0)	111 (81.0)*
HDL-cholesterol (mmol/l)	1.3 ± 0.4	1.1 ± 0.3*	1.3 ± 0.4	1.1 ± 0.3*
Triglycerides (mmol/l)	1.2 (0.8, 1.6)	1.6 (1.1, 1.9)*	1.2 (0.8, 1.6)	1.7 (1.1, 2.1)*
Fasting glucose (mmol/l)	4.5 ± 0.7	7.4 ± 2.2*	4.4 ± 0.6	5.3 ± 0.7*
Lipid-lowering medication [n (%)]	265 (10.3)	99 (46.7)*	136 (9.5)	42 (30.9)*

Data are means ± standard deviations (SD), medians (inter-quartile range), or n (%). Triglycerides were natural logarithm transformed prior to analysis

*p-value <0.05 after adjusting age, sex and/or lipid-lowering medication

### Metabolites associated with type 2 diabetes at baseline

We identified 24 independent metabolites together with five TRFs (age, sex, family history, waist circumference, and HDL-cholesterol) from LASSO regression maximizing the discrimination at baseline. These metabolites and their associations with prevalent and incident type 2 diabetes, as well as fasting glucose at baseline are listed in Table 2. Four of them (i.e. PC(O-34:2), L-HDL-free cholesterol, XXL-LDL-phospholipids and L-LDL-cholesterol) are associated with decreased risk of type 2 diabetes, whereas twenty of them associated with increased risk; including three triglycerides, seven lipoprotein particles, three amino acids, and seven small intermediate compounds. Among the seven lipoprotein particles, two are sub-fractions of HDL, two are of LDL, and three are of VLDL (See details in Table 2). Out of the 24 metabolites, PC(O-34:2), XXL-LDL-triglycerides, HDL-triglycerides, L-HDL-ApoA2, and M-HDL-ApoA2 are not associated with fasting glucose in the non-diabetic population at baseline and incident type 2 diabetes.

**Table 2:** Association of LASSO regression selected metabolites with type 2 diabetes and fasting glucose

Metabolites	ChEBI ID	Prevalent cases versus controls		Incident cases versus controls		Fasting glucose	
		OR [95%CI]	p-value	OR [95%CI]	p-value	Effect	p-value
PC(O-34:2)	CHEBI:64544	0.6 [0.5, 0.7]	1.3 × 10^− 7^	0.9 [0.7, 1.1]	0.19	−0.01	0.28
Isoleucine	CHEBI:24898	2.4 [2.0, 2.9]	2.7 × 10^− 20^	2.0 [1.6, 2.5]	4.4 × 10^− 9^	0.09	3.6 × 10^− 8^
Methionine	CHEBI:16811	1.4 [1.2, 1.6]	1.2 × 10^− 4^	1.3 [1.1, 1.6]	7.4 × 10^− 3^	0.05	2.6 × 10^− 4^
Tyrosine	CHEBI:18186	1.5 [1.2, 1.7]	1.6 × 10^− 5^	2.0 [1.6, 2.5]	5.3 × 10^− 10^	0.13	6.0 × 10^− 18^
2-hydroxybutyrate	CHEBI:64552	2.0 [1.7, 2.5]	2.5 × 10^− 13^	2.0 [1.6, 2.6]	2.6 × 10^− 10^	0.15	2.8 × 10^− 27^
1,5-AG	CHEBI:16070	2.3 [1.9, 2.7]	5.0 × 10^− 19^	1.5 [1.2, 1.8]	3.3 × 10^− 4^	0.09	4.5 × 10^− 10^
2-oxoglutaric acid	CHEBI:30915	1.5 [1.3, 1.8]	2.70 × 10^− 6^	1.8 [1.4, 2.2]	6.0 × 10^− 7^	0.13	8.9 × 10^− 20^
Glycine betaine	CHEBI:17750	2.2 [1.8, 2.6]	2.50 × 10^− 17^	1.5 [1.2, 1.9]	2.3 × 10^− 4^	0.12	1.8 × 10^− 14^
Glycerol	CHEBI:17754	2.3 [1.8, 2.8]	2.1 × 10^− 14^	1.7 [1.3, 2.1]	1.5 × 10^− 5^	0.13	9.1 × 10^− 18^
Lactate	CHEBI:24996	1.7 [1.4, 1.9]	4.9 × 10^− 11^	1.5 [1.2, 1.7]	3.1 × 10^− 5^	0.11	3.1 × 10^− 15^
Pyruvate	CHEBI:15361	1.6 [1.4, 1.8]	3.0 × 10^− 9^	1.5 [1.3, 1.8]	3.3 × 10^− 6^	0.14	1.3 × 10^− 25^
TG (48:0)	CHEBI:85870	1.4 [1.2, 1.6]	2.3 × 10^− 5^	1.6 [1.3, 1.9]	9.3 × 10^− 7^	0.08	2.0 × 10^− 8^
TG (48:1)	CHEBI:85726	1.4 [1.2, 1.6]	1.3 × 10^− 4^	1.5 [1.3, 1.9]	8.0 × 10^− 6^	0.07	5.1 × 10^− 8^
TG (50:5)	CHEBI:90301	1.3 [1.1, 1.4]	2.3 × 10^− 3^	1.5 [1.2, 1.7]	6.5 × 10^− 6^	0.06	1.4 × 10^− 5^
VLDL-free cholesterol	–	1.4 [1.2, 1.7]	7.2 × 10^− 7^	1.6 [1.4, 1.9]	8.2 × 10^− 8^	0.08	1.7 × 10^− 9^
XXL-VLDL-cholesterol	–	1.3 [1.1, 1.5]	4.9 × 10^− 4^	1.5 [1.3, 1.7]	2.9 × 10^− 6^	0.08	1.1 × 10^− 8^
VLDL-triglycerides	–	1.4 [1.2, 1.6]	3.2 × 10^− 6^	1.5 [1.3, 1.8]	1.0 × 10^− 6^	0.09	3.3 × 10^− 10^
XXL-LDL-phospholipids	–	0.6 [0.5, 0.7]	4.4 × 10^− 9^	0.7 [0.6, 0.9]	2.9 × 10^− 3^	−0.06	6.4 × 10^− 5^
XXL-LDL-triglycerides	–	1.4 [1.2, 1.6]	2.4 × 10^− 4^	0.9 [0.7, 1.1]	0.16	0.01	0.34
L-LDL-cholesterol	–	0.5 [0.5, 0.6]	8.1 × 10^− 14^	0.7 [0.6, 0.9]	1.7 × 10^− 3^	−0.05	1.9 × 10^− 4^
XS-LDL-ApoB	–	1.4 [1.2, 1.7]	3.6 × 10^− 6^	1.6 [1.3, 1.9]	3.7 × 10^− 7^	0.05	2.2 × 10^− 4^
L-HDL-ApoA2	–	1.4 [1.2, 1.6]	2.1 × 10^− 4^	1.0 [0.8, 1.2]	0.94	0.02	0.14
L-HDL-free cholesterol	–	0.5 [0.4, 0.6]	3.9 × 10^− 12^	0.7 [0.5, 0.8]	1.5 × 10^− 4^	−0.09	3.2 × 10^− 10^
M-HDL-ApoA2	–	1.4 [1.2, 1.7]	5.8 × 10^− 5^	1.1 [0.9, 1.3]	0.61	0.04	4.1 × 10^− 3^

Odds ratio (OR) and 95% confidence interval (CI) estimates provided from logistic regression and Effect from linear regression with age- sex- and lipid-lowering medication-adjusted in the standardized metabolite variables

### Predicting incident type 2 diabetes

**Fig. 2:**
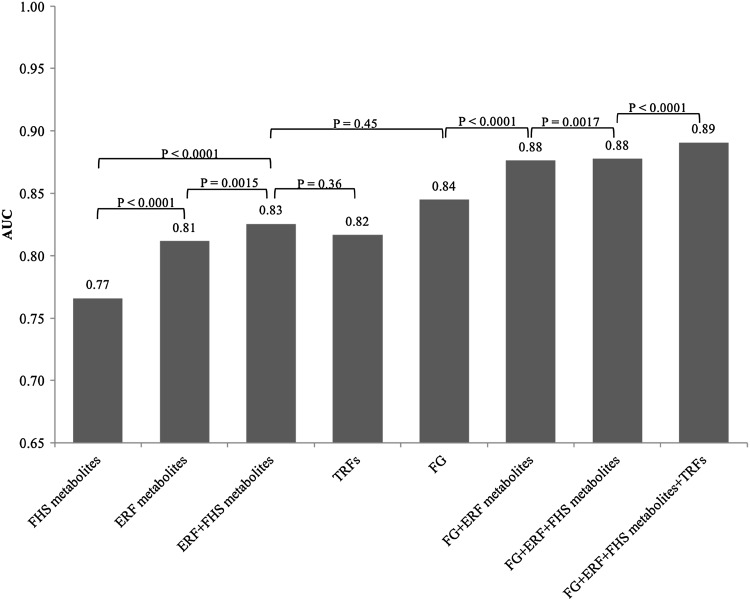
AUC comparisons in different prediction models. Continuous Net Reclassification Improvement (NRI) indices were performed to compare different prediction models. *FG* fasting glucose, *TRFs* all traditional risk factors—age, sex, family history, BMI, waist circumference, hypertension, HDL-cholesterol, triglycerides

Figure 2 shows the AUC comparisons across the different prediction models. The ERF metabolites discriminate future type 2 diabetes with an AUC [95% confidence interval] of 0.81 [0.77, 0.85]. The AUC of the ERF metabolite model was significantly higher than of the FHS metabolite model [AUC 0.81 (0.77, 0.85) vs. 0.77 (0.73, 0.81), NRI = 0.42, p-value <0.0001]. It is of note that tyrosine and isoleucine, which were previously selected by FHS, were also selected in the ERF metabolite model. The AUC for the model including both ERF and FHS metabolites together was significantly higher than the AUC for models with either set of predictors [AUC 0.83 (0.79, 0.86) vs. 0.77 (0.73, 0.81), NRI = 0.67, p-value <0.0001 for ERF and FHS metabolites vs. only FHS metabolites; AUC 0.83 (0.79, 0.86) vs. 0.81 (0.77, 0.85), NRI = 0.29, p-value =0.0015 for ERF and FHS metabolites vs. only ERF metabolites]. The AUC of the ERF and FHS combined metabolite model did not differ from that of fasting glucose [AUC 0.83 (0.79, 0.86) vs. 0.84 (0.81, 0.88), p-value =0.45]. However, combining the ERF metabolites and fasting glucose together in a model improved the predictive performance significantly over the performance of fasting glucose [AUC 0.88 (0.84, 0.91) vs. 0.84 (0.81, 0.88), NRI = 0.66, p-value <0.0001]. Adding TRFs to fasting glucose and metabolite model maximized the AUC to 0.89 [0.86, 0.92]. The specificity with fixed 80% sensitivity increases from 70 to 80% when metabolites are added to the glucose only model (Supplementary Fig. 1).

### Predicting incident type 2 diabetes in different baseline risk groups

**Fig. 3:**
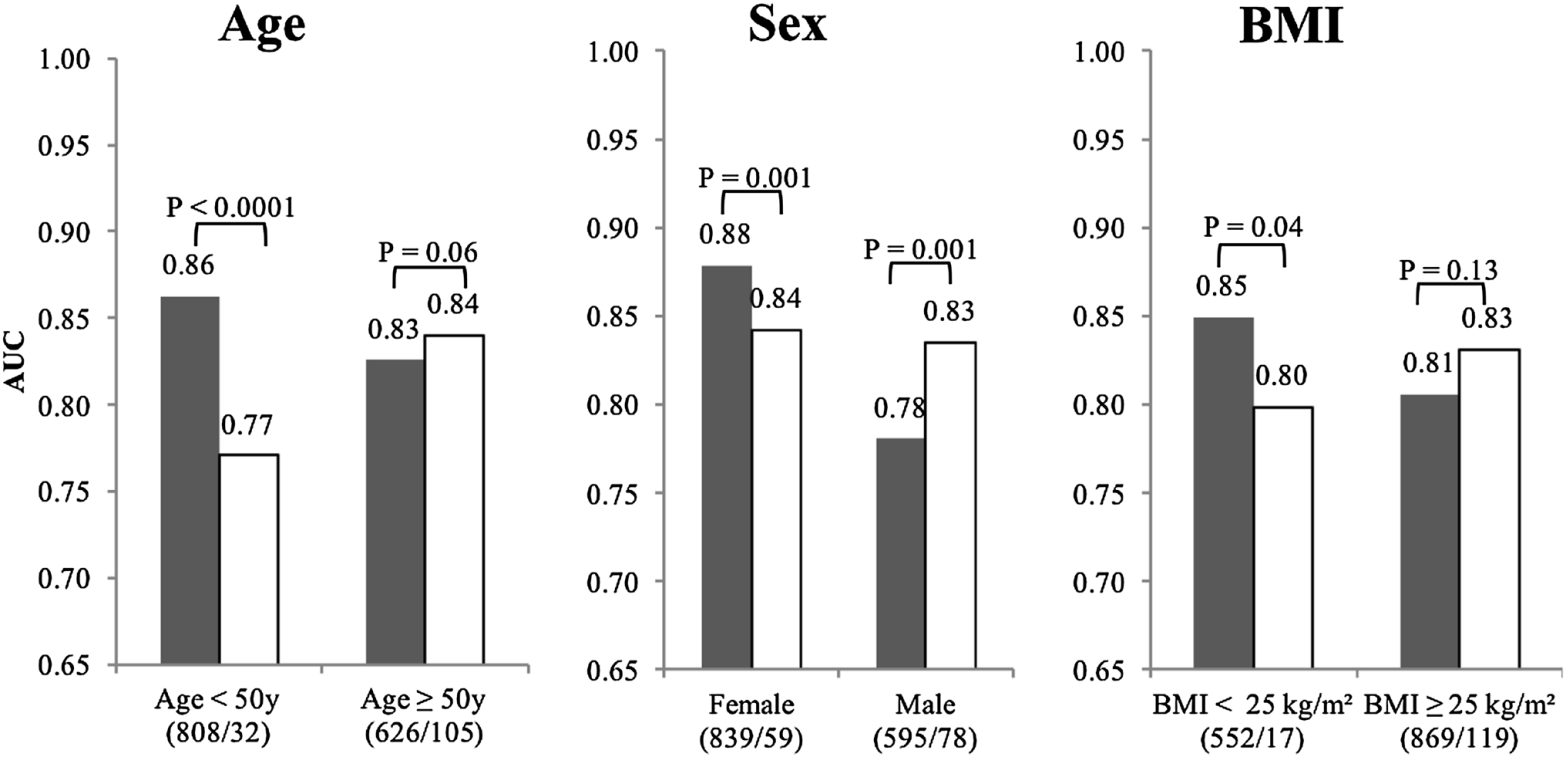
AUC comparisons in different subgroups. Continuous Net Reclassification Improvement (NRI) indices were performed to compare different prediction models. *Black bars* metabolite model; *white bars* fasting glucose model. (/): Number of controls and incident cases analyzed in the follow-up

The AUC of the combined ERF, and FHS metabolite models and of fasting glucose model in subpopulations stratified by age, sex, and BMI is shown in Fig. 3. In the group with age < 50 years, the AUC of the combined metabolite model is significantly higher than that of fasting glucose model [AUC 0.86 (0.78, 0.94) vs. 0.77 (0.67, 0.87), NRI = 0.72, p-value <0.0001], whereas the AUCs of these two models are not statistically different in the elderly group [AUC 0.83 (0.78, 0.87) vs. 0.84 (0.80, 0.88), p-value =0.06]. The AUC of the metabolite model is significantly higher than that of fasting glucose in the female group [AUC 0.88 (0.83, 0.92) vs. 0.84 (0.79, 0.90), NRI = 0.44, p-value =0.001], whereas in the male group there is an opposite trend (0.78 [0.72, 0.84] vs. 0.83 [0.79, 0.88], NRI = −0.40, p-value =0.001). Similarly, in the group with normal BMI, the AUC of metabolite model is significantly higher than that of fasting glucose model [AUC 0.85 (0.75, 0.95) vs. 0.80 (0.66, 0.93), NRI = 0.49, p-value =0.04]. In the overweight and obese group, the trend is opposite but not significantly different [AUC 0.81 (0.76, 0.85) vs. 0.83 (0.79, 0.87), p-value =0.13]. When the sensitivity is fixed to 80%, the specificity rises from 59% (glucose only model) to 87% (glucose and metabolite model) in the young (age < 50 years), which is much higher increase than in the old (age ≥ 50 years, from 66 to 82%). The specificity also grows when we add metabolites or TRFs to the prediction model. (Supplementary Fig. 1) The ROC curves for the models and subgroups are given in the Supplementary Fig. 2 and Supplementary Fig. 3. The separation shown by time to event curves across different risk groups are given in Supplementary Fig. 4.

## Discussion

In the present study, we showed that the combined effect of 24 metabolites including ten lipoprotein sub-fractions yield a powerful discrimination model for predicting future type 2 diabetes. The ERF metabolite model significantly improved the prediction performance of FHS metabolite model and fasting glucose. We showed that combined metabolite model predicts future type 2 diabetes better than fasting glucose in the population who are female, younger than 50 years, or those with normal weight. In addition, we confirmed the conclusion from the FHS that isoleucine and tyrosine are predictors of type 2 diabetes independent of other factors (Wang et al. 2011).

The ERF metabolite model includes molecules from five classes: triglycerides, amino acids, lipoproteins, phospholipids and small intermediate compounds. Among those, metabolites such as 1,5-anhydro-d-glucitol (1,5-AG), 2-hydroxybutyrate, pyruvate, phosphatidylcholines, betaine, some triglycerides, and BCAA have been previously reported to be potential predictive and diagnostic markers for type 2 diabetes. (Wang et al. 2011; Nanditha et al. 2014; Wang-Sattler et al. 2012; Kim et al. 2016; Park et al. 2015; Yousri et al. 2015). Despite the fact that LASSO regression method is used to select independent components of our model, various metabolites from the same biochemical class were selected, supporting the view that the sub-fractions of some classical measurements play independent functions in the pathogenesis of type 2 diabetes (Kotronen et al. 2009). In line with this, the ERF metabolite model points out lipid perturbations evident in the very early stage of the disease. For example, levels of different triglycerides [e.g. TG (48:0), TG (48:1)] show independent effects. Our results on HDL and LDL sub-fractions are particularly interesting. We found them associated with both increased and decreased risk. L-HDL-ApoA2, M-HDL-ApoA2, XS-LDL-ApoB and XXL-LDL-triglycerides are associated with increased risk of type 2 diabetes, whereas L-HDL-free cholesterol, XXL-LDL-phospholipids and L-LDL-cholesterol are associated with decreased risk of type 2 diabetes. This suggests different roles for HDL and LDL particles and their content. Our results highlight the importance of reclassifying lipoproteins of clinical value into sub-fractions of HDL, LDL and VLDL, as the measurement techniques develop in the coming decade.

We also demonstrated that PC(O-34:2) is inversely associated with type 2 diabetes, which is in line with a recent study performed in the population based KORA study that showed decrease in PC(O-34:2) levels in patients with impaired glucose tolerance (Wang-Sattler et al. 2012). Phosphatidylcholine is a key element in lipoproteins (Park et al. 2015). Elevated plasma levels of choline and betaine mark cardiovascular risk in diabetes (Lever et al. 2014), while increased level of isoleucine was significantly associated with an increased risk of hypertriglyceridemia (Mook-Kanamori et al. 2014). 2-hydroxybutyrate appears to be useful as an early indicator of insulin resistance in non-diabetic subjects (Gall et al. 2010), and its elevated serum levels have recently been indicated to predict worsening of glucose tolerance (Ferrannini et al. 2013).

Among the other ERF metabolites, our results on two (1,5-AG and glycerol) are inconsistent with the previous studies in terms of direction of association: Suhre et al. studied on 40 diabetes cases and 60 healthy male controls in the German population (Suhre et al. 2010); Lu J. et al.’s study included 22 Chinese cases and 22 healthy controls (Lu et al. 2012), and the study by Shaham O. et al. was done in 47 healthy academic students (Shaham et al. 2008). Considering the larger sample size, our study should have yielded more reliable estimates compared to the above studies. It has been shown that levels of 1,5-AG metabolite reflect glycemic changes, and recent clinical studies demonstrated significant differences in 1,5-AG levels between diabetic patients receiving different treatments, consistent with their individual glucose profiles (McGill et al. 2004; Moses et al. 2008).

As shown in Table 2, all metabolites are associated with prevalent diabetes, but some are not associated with incident case control status. We kept those in the ERF prediction model as this could be due to their small effect sizes which need more sample size (power) to be detected since the effect estimates were in the expected direction. Another explanation could be that some metabolite levels change depending on the duration and progression of the disease that we cannot control for in the statistical model. A third explanation is that it could as well be due to anti-diabetic medication effect but we have already filtered the associations controlling for that. The addition of ERF metabolites can complement the type 2 diabetes prediction by fasting glucose and TRFs, yielding the best model when combined. This is partly a result of our selection approach performed independently of TRFs but may also be due to the assumption that high resolution metabolites reflect different possible etiologies of type 2 diabetes. Thus, improvement of predictive performance with additional metabolites implies that potential metabolic ramifications may extend far beyond and prior to impaired glucose metabolism. It is of note that each metabolite contributed equally to the improvement of the AUC except tyrosine, exclusion of which dropped the AUC significantly. The AUC of the model without tyrosine is 0.79, and is significantly lower than the AUC of ERF metabolite model which is 0.81 (NRI = 0.38, p-value <0.0001), suggesting that tyrosine is an important component of the model.

In the present study, we found a higher AUC of the metabolite model in lower risk population as female, younger, or leaner subgroups. For the optimum cut-off value of the ROC curve, we observed the biggest difference in specificity especially in the young age group, such that if the sensitivity of the prediction model is set to 80%, the metabolite only model yielded a specificity of 0.82, whereas the glucose model is as low as 0.59. This suggests that the metabolomics information may have better utility for type 2 diabetes prediction specifically in those without the risk condition, which is in agreement with previous study from Walford et al.(Walford et al. 2014). Interestingly, low risk population that develop type 2 diabetes were reported to have higher risk of mortality (Carnethon et al. 2012), raising the importance of more specific predictors suited for different underlying mechanisms. Markers which reflect the metabolic condition both dependent and independent of BMI that may partially help to address the different active pathways underlying to the MUHNW and MHO phenotypes (Mathew et al. 2016).

Two additional platforms measured among subsets of the ERF population which were not included in our main analysis due to sample size restrictions gave us the opportunity to compare some of the associations using these different measurement methods. These were electrospray-Ionization MS, measured in 878 participants, using the method described before (Demirkan et al. 2012) and AbsoluteIDQTM p150 Kit of Biocrates Life Sciences AG measured in 989 participants as details mentioned before (Draisma et al. 2015). Supplementary Fig. 5 shows the x–y plots of the effect estimates per standard error (i.e. Z score) in the 62 lipids and 9 amino acids that were measured in duplication. The Z scores between these platforms are strongly correlated with correlation coefficients ranging from 0.74 to 0.87.

The present study has a strong design such that the new cases develop among the control group in the baseline. However, due to the wide metabolite spectrum in the present study, validation of the full model in an external sample is not available yet. One limitation can be that in the present study, 46.7% of the type 2 diabetes patients at baseline took lipid-lowering medication compared 10.3% in the non-diabetics. To reduce the bias, all the participants were fasted overnight before taking the blood sample and we adjusted for lipid-lowering medication in each step of statistical analysis. It also needs mentioning that the metabolite set that predicts type 2 diabetes is assumed to point out the biochemical pathways disrupted before the disease onset. However, these metabolites may not be necessarily in the causal pathway. We have previously shown that most these metabolites are partially heritable (Demirkan et al. 2015; Kettunen et al. 2016; Draisma et al. 2015) and our increasing knowledge about their genetic determinants opens up new opportunities for testing causal inference using Mendelian randomization (Kettunen et al. 2016).

Conducting a 14-years prospective study with comparably large sample size and wide metabolite spectrum, we developed a novel prediction model which includes informative markers of dyslipidemia, and which also increases the specificity for the young individuals. Importantly, this model has a high potential to result with better understanding of the biological mechanisms leading to glycemic deterioration in prediabetes and diabetes.

## Electronic supplementary material

Below is the link to the electronic supplementary material.

Supplementary material 1 (DOCX 36 KB)

Supplementary material 2 (DOCX 1760 KB)

Supplementary material 3 (DOCX 758 KB)

## References

[CR1] Andrew G, Jennifer H (2006). Data analysis using regression and multilevel/hierarchical models.

[CR2] Aulchenko YS, de Koning DJ, Haley C (2007). Genomewide rapid association using mixed model and regression: a fast and simple method for genomewide pedigree-based quantitative trait loci association analysis. Genetics.

[CR3] Carnethon MR, De Chavez PJ, Biggs ML, Lewis CE, Pankow JS, Bertoni AG (2012). Association of weight status with mortality in adults with incident diabetes. JAMA.

[CR4] Demirkan A, Henneman P, Verhoeven A, Dharuri H, Amin N, van Klinken JB (2015). Insight in genome-wide association of metabolite quantitative traits by exome sequence analyses. PLoS Genetics.

[CR5] Demirkan A, van Duijn CM, Ugocsai P, Isaacs A, Pramstaller PP, Liebisch G (2012). Genome-wide association study identifies novel loci associated with circulating phospho- and sphingolipid concentrations. PLoS Genetics.

[CR6] Draisma HH, Pool R, Kobl M, Jansen R, Petersen AK, Vaarhorst AA (2015). Genome-wide association study identifies novel genetic variants contributing to variation in blood metabolite levels. Nature Communications.

[CR7] Droumaguet C, Balkau B, Simon D, Caces E, Tichet J, Charles MA (2006). Use of HbA1c in predicting progression to diabetes in French Men and Women data from an Epidemiological Study on the Insulin Resistance Syndrome (DESIR). Diabetes Care.

[CR8] Dunn WB, Lin W, Broadhurst D, Begley P, Brown M, Zelena E (2015). Molecular phenotyping of a UK population: Defining the human serum metabolome. Metabolomics.

[CR9] Ferrannini E, Natali A, Camastra S, Nannipieri M, Mari A, Adam KP (2013). Early metabolic markers of the development of dysglycemia and type 2 diabetes and their physiological significance. Diabetes.

[CR10] Floegel A, Stefan N, Yu Z, Muhlenbruch K, Drogan D, Joost HG (2013). Identification of serum metabolites associated with risk of type 2 diabetes using a targeted metabolomic approach. Diabetes.

[CR11] Friedman J, Hastie T, Tibshirani R (2010). Regularization paths for generalized linear models via coordinate descent. Journal of statistical software.

[CR12] Gall WE, Beebe K, Lawton KA, Adam KP, Mitchell MW, Nakhle PJ (2010). alpha-hydroxybutyrate is an early biomarker of insulin resistance and glucose intolerance in a nondiabetic population. PLoS ONE.

[CR13] Gonzalez-Covarrubias V, Beekman M, Uh HW, Dane A, Troost J, Paliukhovich I (2013). Lipidomics of familial longevity. Aging Cell.

[CR14] Gray LJ, Taub NA, Khunti K, Gardiner E, Hiles S, Webb DR (2010). The Leicester Risk Assessment score for detecting undiagnosed type 2 diabetes and impaired glucose regulation for use in a multiethnic UK setting. Diabetic Medicine: A Journal of the British Diabetic Association.

[CR15] Haffner SM, Stern MP, Mitchell BD, Hazuda HP, Patterson JK (1990). Incidence of type II diabetes in Mexican Americans predicted by fasting insulin and glucose levels, obesity, and body-fat distribution. Diabetes.

[CR16] Haug K, Salek RM, Conesa P, Hastings J, de Matos P, Rijnbeek M (2013). MetaboLights–an open-access general-purpose repository for metabolomics studies and associated meta-data. Nucleic Acids Research.

[CR17] Hu C, van Dommelen J, van der Heijden R, Spijksma G, Reijmers TH, Wang M (2008). RPLC-ion-trap-FTMS method for lipid profiling of plasma: method validation and application to p53 mutant mouse model. Journal of Proteome Research.

[CR18] Kengne AP, Beulens JW, Peelen LM, Moons KG, van der Schouw YT, Schulze MB (2014). Non-invasive risk scores for prediction of type 2 diabetes (EPIC-InterAct): A validation of existing models. Lancet Diabetes Endocrinol.

[CR19] Kettunen J, Demirkan A, Wurtz P, Draisma HH, Haller T, Rawal R (2016). Genome-wide study for circulating metabolites identifies 62 loci and reveals novel systemic effects of LPA. Nature Communications.

[CR20] Kim YJ, Lee HS, Kim YK, Park S, Kim JM, Yun JH (2016). Association of metabolites with obesity and type 2 diabetes based on FTO genotype. PLoS ONE.

[CR21] Knowler WC, Barrett-Connor E, Fowler SE, Hamman RF, Lachin JM, Walker EA (2002). Reduction in the incidence of type 2 diabetes with lifestyle intervention or metformin. The New England Journal of Medicine.

[CR22] Kotronen A, Velagapudi VR, Yetukuri L, Westerbacka J, Bergholm R, Ekroos K (2009). Serum saturated fatty acids containing triacylglycerols are better markers of insulin resistance than total serum triacylglycerol concentrations. Diabetologia.

[CR23] Lawton KA, Berger A, Mitchell M, Milgram KE, Evans AM, Guo L (2008). Analysis of the adult human plasma metabolome. Pharmacogenomics.

[CR24] Lever M, George PM, Slow S, Bellamy D, Young JM, Ho M (2014). Betaine and trimethylamine-N-oxide as predictors of cardiovascular outcomes show different patterns in diabetes mellitus: an observational study. PLoS ONE.

[CR25] Li J, Ji L (2005). Adjusting multiple testing in multilocus analyses using the eigenvalues of a correlation matrix. Heredity (Edinb).

[CR26] Li R, Zhang P, Barker LE, Chowdhury FM, Zhang X (2010). Cost-effectiveness of interventions to prevent and control diabetes mellitus: A systematic review. Diabetes care.

[CR27] Lu J, Zhou J, Bao Y, Chen T, Zhang Y, Zhao A (2012). Serum metabolic signatures of fulminant type 1 diabetes. Journal of Proteome Research.

[CR28] Lu Y, Wang Y, Ong CN, Subramaniam T, Choi HW, Yuan JM (2016). Metabolic signatures and risk of type 2 diabetes in a Chinese population: an untargeted metabolomics study using both LC-MS and GC-MS. Diabetologia.

[CR29] Mathew H, Farr OM, Mantzoros CS (2016). Metabolic health and weight: Understanding metabolically unhealthy normal weight or metabolically healthy obese patients. Metabolism: Clinical and Experimental.

[CR30] McGill JB, Cole TG, Nowatzke W, Houghton S, Ammirati EB, Gautille T (2004). Circulating 1,5-anhydroglucitol levels in adult patients with diabetes reflect longitudinal changes of glycemia: A U.S. trial of the GlycoMark assay. Diabetes Care.

[CR31] Mook-Kanamori DO, Romisch-Margl W, Kastenmuller G, Prehn C, Petersen AK, Illig T (2014). Increased amino acids levels and the risk of developing of hypertriglyceridemia in a 7-year follow-up. Journal of Endocrinological Investigation.

[CR32] Moses AC, Raskin P, Khutoryansky N (2008). Does serum 1,5-anhydroglucitol establish a relationship between improvements in HbA1c and postprandial glucose excursions? Supportive evidence utilizing the differential effects between biphasic insulin aspart 30 and insulin glargine. Diabetic Medicine: A Journal of the British Diabetic Association.

[CR33] Nanditha A, Ram J, Snehalatha C, Selvam S, Priscilla S, Shetty AS (2014). Early improvement predicts reduced risk of incident diabetes and improved cardiovascular risk in prediabetic Asian Indian men participating in a 2-year lifestyle intervention program. Diabetes Care.

[CR34] Park S, Sadanala KC, Kim EK (2015). A metabolomic approach to understanding the metabolic link between obesity and diabetes. Molecules and Cells.

[CR35] Pencina MJ, D’Agostino RB, Demler OV (2012). Novel metrics for evaluating improvement in discrimination: Net reclassification and integrated discrimination improvement for normal variables and nested models. Statistics in Medicine.

[CR36] Sansone SA, Fan T, Goodacre R, Griffin JL, Hardy NW, Kaddurah-Daouk R (2007). The metabolomics standards initiative. Nature Biotechnology.

[CR37] Santos RL, Zillikens MC, Rivadeneira FR, Pols HA, Oostra BA, van Duijn CM (2006). Heritability of fasting glucose levels in a young genetically isolated population. Diabetologia.

[CR38] Shaham O, Wei R, Wang TJ, Ricciardi C, Lewis GD, Vasan RS (2008). Metabolic profiling of the human response to a glucose challenge reveals distinct axes of insulin sensitivity. Molecular Systems Biology.

[CR39] Shaw JE, Zimmet PZ, de Courten M, Dowse GK, Chitson P, Gareeboo HA (1999). Impaired fasting glucose or impaired glucose tolerance. What best predicts future diabetes in Mauritius?. Diabetes Care.

[CR40] Suhre K, Meisinger C, Doring A, Altmaier E, Belcredi P, Gieger C (2010). Metabolic footprint of diabetes: A multiplatform metabolomics study in an epidemiological setting. PLoS ONE.

[CR41] Tabak AG, Jokela M, Akbaraly TN, Brunner EJ, Kivimaki M, Witte DR (2009). Trajectories of glycaemia, insulin sensitivity, and insulin secretion before diagnosis of type 2 diabetes: An analysis from the Whitehall II study. Lancet.

[CR42] Verhoeven A, Slagboom E, Wuhrer M, Giera M, Mayboroda OA (2017). Automated quantification of metabolites in blood-derived samples by NMR. Analytica Chimica Acta.

[CR43] Walford GA, Porneala BC, Dauriz M, Vassy JL, Cheng S, Rhee EP (2014). Metabolite traits and genetic risk provide complementary information for the prediction of future type 2 diabetes. Diabetes Care.

[CR44] Wang TJ, Larson MG, Vasan RS, Cheng S, Rhee EP, McCabe E (2011). Metabolite profiles and the risk of developing diabetes. Natural Medicines.

[CR45] Wang-Sattler R, Yu Z, Herder C, Messias AC, Floegel A, He Y (2012). Novel biomarkers for pre-diabetes identified by metabolomics. Molecular Systems Biology.

[CR46] Wilson PW, Meigs JB, Sullivan L, Fox CS, Nathan DM, D’Agostino RB (2007). Prediction of incident diabetes mellitus in middle-aged adults: The Framingham Offspring Study. Archives of Internal Medicine.

[CR47] Yousri NA, Mook-Kanamori DO, Selim MM, Takiddin AH, Al-Homsi H, Al-Mahmoud KA (2015). A systems view of type 2 diabetes-associated metabolic perturbations in saliva, blood and urine at different timescales of glycaemic control. Diabetologia.

[CR48] Yu D, Moore SC, Matthews CE, Xiang YB, Zhang X, Gao YT (2016). Plasma metabolomic profiles in association with type 2 diabetes risk and prevalence in Chinese adults. Metabolomics.

