# Supplementary material for: Metabolomics based markers predict type 2 diabetes in a 14-year follow-up study
Source: Metabolomics. 2017 Jul 28;13(9):104. doi: 10.1007/s11306-017-1239-2 (PMC5533833; doi:10.1007/s11306-017-1239-2)
Supplement: Supplementary file 1 — Supplementary material 1 (DOCX 36 KB) [file 11306_2017_1239_MOESM1_ESM.docx]

**Supplementary Table 1** Metabolites which are selected to be included in LASSO regression and their association with type 2 diabetes and fasting glucose

|  |  | ChEBI ID | Prevalent cases *vs* controls | | Incident cases *vs* controls | | Fasting glucose | |
| --- | --- | --- | --- | --- | --- | --- | --- | --- |
| Platform | Metabolites |  | OR [95%CI] | P | OR [95%CI] | P-value | Effect | P |
| LC-MS | PC(O-34:1) | CHEBI:67076 | 0.64[0.53,0.77] | 3.40E-06 | 0.63[0.5,0.79] | 1.20E-04 | -0.09 | 1.50E-11 |
| LC-MS | PC(O-34:2) | CHEBI:64544 | 0.57[0.45,0.69] | 1.30E-07 | 0.87[0.7,1.07] | 1.90E-01 | -0.01 | 2.80E-01 |
| LC-MS | PC(O-34:3) | CHEBI:64541 | 0.59[0.48,0.72] | 3.40E-07 | 0.59[0.46,0.74] | 1.70E-05 | -0.07 | 4.70E-07 |
| LC-MS | PC(O-36:2) | CHEBI:67065 | 0.56[0.45,0.68] | 2.90E-08 | 0.65[0.51,0.82] | 2.70E-04 | -0.08 | 1.10E-08 |
| NMR-COMP | Alanine | CHEBI:16449 | 1.49[1.26,1.76] | 3.40E-06 | 1.82[1.48,2.24] | 1.50E-08 | 0.12 | 2.00E-17 |
| NMR-COMP | Isoleucine | CHEBI:24898 | 2.39[1.99,2.89] | 2.70E-20 | 1.95[1.56,2.45] | 4.40E-09 | 0.09 | 3.60E-08 |
| NMR-COMP | Leucine | CHEBI:25017 | 2.34[1.95,2.83] | 1.90E-19 | 1.92[1.54,2.41] | 1.40E-08 | 0.09 | 4.30E-08 |
| NMR-COMP | Lysine | CHEBI:25094 | 1.52[1.26,1.84] | 1.30E-05 | 1.17[0.94,1.48] | 1.70E-01 | 0.05 | 1.60E-03 |
| NMR-COMP | Methionine | CHEBI:16811 | 1.39[1.18,1.64] | 0.00012 | 1.32[1.08,1.62] | 7.40E-03 | 0.05 | 2.60E-04 |
| NMR-COMP | Phenylalanine | CHEBI:28044 | 1.72[1.45,2.04] | 5.40E-10 | 1.75[1.41,2.17] | 3.20E-07 | 0.09 | 6.80E-10 |
| NMR-COMP | Tyrosine | CHEBI:18186 | 1.46[1.23,1.74] | 1.60E-05 | 1.99[1.61,2.49] | 5.30E-10 | 0.13 | 6.00E-18 |
| NMR-COMP | Valine | CHEBI:27266 | 2.32[1.94,2.78] | 3.80E-20 | 2.11[1.69,2.65] | 7.50E-11 | 0.13 | 4.90E-17 |
| NMR-COMP | 2-oxoglutaric acid | CHEBI:30915 | 1.53[1.29,1.84] | 2.70E-06 | 1.76[1.41,2.2] | 6.00E-07 | 0.13 | 8.90E-20 |
| NMR-COMP | 3-methyl-2-oxobutanoic acid | CHEBI:16530 | 1.92[1.63,2.26] | 1.10E-14 | 1.99[1.61,2.46] | 3.00E-10 | 0.13 | 3.50E-21 |
| NMR-COMP | 2-hydroxybutyrate | CHEBI:64552 | 2.04[1.69,2.47] | 2.50E-13 | 2.04[1.64,2.56] | 2.60E-10 | 0.15 | 2.80E-27 |
| NMR-COMP | 3-hydroxyisobutyrate | CHEBI:11805 | 1.57[1.35,1.82] | 2.10E-09 | 1.66[1.39,1.99] | 2.90E-08 | 0.13 | 1.50E-20 |
| NMR-COMP | 1,5-anhydro-D-glucitol | CHEBI:16070 | 2.26[1.89,2.71] | 5.00E-19 | 1.48[1.2,1.84] | 3.30E-04 | 0.09 | 4.50E-10 |
| NMR-COMP | Acetone | CHEBI:15347 | 1.82[1.52,2.18] | 6.80E-11 | 1.58[1.27,1.97] | 4.60E-05 | 0.04 | 1.10E-02 |
| NMR-COMP | Glycine betaine | CHEBI:17750 | 2.19[1.83,2.63] | 2.50E-17 | 1.5[1.21,1.87] | 2.30E-04 | 0.12 | 1.80E-14 |
| NMR-COMP | Glycerol | CHEBI:17754 | 2.25[1.83,2.78] | 2.10E-14 | 1.67[1.33,2.11] | 1.50E-05 | 0.13 | 9.10E-18 |
| NMR-COMP | Lactate | CHEBI:24996 | 1.65[1.42,1.91] | 4.90E-11 | 1.45[1.22,1.73] | 3.10E-05 | 0.11 | 3.10E-15 |
| NMR-COMP | Pyruvate | CHEBI:15361 | 1.56[1.35,1.81] | 3.00E-09 | 1.53[1.28,1.83] | 3.30E-06 | 0.14 | 1.30E-25 |
| NMR-COMP | Lipids (CH=CH*CH2CH2) | - | 1.42[1.19,1.7] | 0.00015 | 1.7[1.35,2.15] | 6.80E-06 | 0.07 | 3.00E-07 |
| NMR-COMP | Lipids (CH2) | - | 1.5[1.25,1.8] | 1.60E-05 | 1.74[1.38,2.21] | 3.10E-06 | 0.08 | 8.20E-08 |
| NMR-COMP | Lipids-CH2CO | - | 1.52[1.27,1.83] | 6.50E-06 | 1.77[1.42,2.24] | 9.10E-07 | 0.08 | 3.40E-08 |
| NMR-COMP | Lipids-CH3 | - | 1.5[1.25,1.8] | 1.20E-05 | 1.65[1.32,2.09] | 1.70E-05 | 0.07 | 3.00E-06 |
| LC-MS | TG(48:0) | CHEBI:85870 | 1.38[1.19,1.59] | 2.30E-05 | 1.56[1.31,1.87] | 9.30E-07 | 0.08 | 2.00E-08 |
| LC-MS | TG(48:1) | CHEBI:85726 | 1.36[1.16,1.59] | 0.00013 | 1.54[1.28,1.87] | 8.00E-06 | 0.07 | 5.10E-08 |
| LC-MS | TG(50:0) | CHEBI:85874 | 1.42[1.2,1.68] | 4.40E-05 | 1.6[1.3,1.99] | 1.10E-05 | 0.07 | 1.00E-06 |
| LC-MS | TG(50:1) | CHEBI:84665 | 1.46[1.22,1.75] | 3.90E-05 | 1.91[1.52,2.42] | 4.50E-08 | 0.06 | 1.50E-06 |
| LC-MS | TG(50:2) | CHEBI:84662 | 1.38[1.16,1.65] | 0.00035 | 1.74[1.39,2.19] | 1.90E-06 | 0.06 | 1.50E-05 |
| LC-MS | TG(50:5) | CHEBI:90301 | 1.25[1.08,1.44] | 0.0023 | 1.46[1.24,1.72] | 6.50E-06 | 0.06 | 1.40E-05 |
| LC-MS | TG(52:1) | CHEBI:90302 | 1.53[1.28,1.82] | 2.10E-06 | 1.76[1.41,2.2] | 8.10E-07 | 0.07 | 7.50E-07 |
| LC-MS | TG(52:2) | CHEBI:85736 | 1.42[1.19,1.7] | 0.00016 | 1.76[1.4,2.24] | 2.50E-06 | 0.04 | 4.30E-03 |
| LC-MS | TG(52:3) | CHEBI:84661 | 1.39[1.17,1.67] | 3.00E-04 | 1.61[1.29,2.03] | 3.90E-05 | 0.03 | 1.50E-02 |
| LC-MS | TG(52:6) | CHEBI:85875 | 1.38[1.17,1.63] | 0.00012 | 1.62[1.32,2] | 4.30E-06 | 0.06 | 2.30E-05 |
| LC-MS | TG(54:1) | CHEBI:90305 | 1.29[1.12,1.48] | 0.00025 | 1.53[1.3,1.79] | 1.40E-07 | 0.07 | 5.60E-07 |
| LC-MS | TG(54:2) | CHEBI:85743 | 1.39[1.17,1.65] | 0.00019 | 1.55[1.24,1.94] | 1.10E-04 | 0.04 | 8.70E-03 |
| LC-MS | TG(54:7) | CHEBI:85740 | 1.31[1.14,1.51] | 0.00016 | 1.55[1.31,1.83] | 3.40E-07 | 0.06 | 8.80E-06 |
| LC-MS | TG(56:7) | CHEBI:85749 | 1.36[1.15,1.61] | 0.00046 | 1.66[1.34,2.08] | 5.50E-06 | 0.04 | 1.90E-03 |
| LC-MS | TG(56:8) | CHEBI:85748 | 1.31[1.13,1.51] | 0.00037 | 1.62[1.36,1.94] | 8.20E-08 | 0.05 | 4.80E-05 |
| LC-MS | TG(58:10) | CHEBI:85752 | 1.29[1.12,1.47] | 0.00035 | 1.41[1.18,1.67] | 8.60E-05 | 0.05 | 3.00E-04 |
| NMR-LIPO | Plasma-triglycerides | - | 1.47[1.25,1.74] | 4.80E-06 | 1.61[1.31,1.99] | 8.60E-06 | 0.06 | 4.50E-06 |
| NMR-LIPO | VLDL-ApoB | - | 1.47[1.27,1.69] | 1.60E-07 | 1.57[1.32,1.87] | 3.00E-07 | 0.09 | 3.40E-10 |
| NMR-LIPO | VLDL-free cholesterol | - | 1.43[1.24,1.65] | 7.20E-07 | 1.6[1.35,1.9] | 8.20E-08 | 0.08 | 1.70E-09 |
| NMR-LIPO | VLDL-phospholipids | - | 1.35[1.17,1.56] | 2.80E-05 | 1.6[1.35,1.89] | 3.80E-08 | 0.08 | 8.60E-09 |
| NMR-LIPO | VLDL-triglycerides | - | 1.39[1.21,1.6] | 3.20E-06 | 1.54[1.29,1.83] | 1.00E-06 | 0.09 | 3.30E-10 |
| NMR-LIPO | XXL-VLDL-cholesterol | - | 1.29[1.11,1.48] | 0.00049 | 1.48[1.25,1.74] | 2.90E-06 | 0.08 | 1.10E-08 |
| NMR-LIPO | XXL-VLDL-free cholesterol | - | 1.36[1.15,1.61] | 0.00041 | 1.64[1.33,2.04] | 5.60E-06 | 0.07 | 2.70E-07 |
| NMR-LIPO | XL-VLDL-cholesterol | - | 1.3[1.13,1.5] | 0.00029 | 1.53[1.29,1.82] | 8.40E-07 | 0.06 | 2.00E-06 |
| NMR-LIPO | L-VLDL-triglycerides | - | 1.45[1.19,1.78] | 0.00025 | 1.6[1.26,2.08] | 2.30E-04 | 0.05 | 9.30E-04 |
| NMR-LIPO | M-VLDL-phospholipids | - | 1.35[1.16,1.57] | 8.40E-05 | 1.41[1.17,1.69] | 2.10E-04 | 0.05 | 1.60E-04 |
| NMR-LIPO | M-VLDL-triglycerides | - | 1.47[1.27,1.7] | 3.60E-07 | 1.51[1.26,1.81] | 5.50E-06 | 0.06 | 7.50E-06 |
| NMR-LIPO | XS-VLDL-phospholipids | - | 1.39[1.17,1.66] | 0.00017 | 1.59[1.28,1.99] | 3.20E-05 | 0.07 | 6.40E-08 |
| NMR-LIPO | XS-VLDL-triglycerides | - | 2.47[2.04,3] | 4.00E-20 | 1.76[1.42,2.2] | 4.80E-07 | 0.15 | 1.10E-29 |
| NMR-LIPO | IDL-phospholipids | - | 1.34[1.14,1.57] | 0.00032 | 1.56[1.28,1.89] | 6.40E-06 | 0.07 | 1.70E-06 |
| NMR-LIPO | IDL-triglycerides | - | 0.98[0.87,1.23] | 0.86 | 2.16[1.58,2.98] | 1.80E-06 | 0.07 | 4.30E-08 |
| NMR-LIPO | LDL-triglycerides | - | 1.55[1.33,1.81] | 1.90E-08 | 1.43[1.18,1.71] | 1.90E-04 | 0.06 | 2.50E-05 |
| NMR-LIPO | XXL-LDL-cholesterol | - | 0.58[0.48,0.7] | 1.20E-08 | 0.72[0.58,0.9] | 3.80E-03 | -0.05 | 9.30E-04 |
| NMR-LIPO | XXL-LDL-free cholesterol | - | 0.59[0.49,0.71] | 3.40E-08 | 0.71[0.57,0.88] | 2.00E-03 | -0.05 | 7.60E-04 |
| NMR-LIPO | XXL-LDL-phospholipids | - | 0.57[0.47,0.68] | 4.40E-09 | 0.72[0.58,0.89] | 2.90E-03 | -0.06 | 6.40E-05 |
| NMR-LIPO | XXL-LDL-triglycerides | - | 1.36[1.16,1.61] | 0.00024 | 0.86[0.7,1.06] | 1.60E-01 | 0.01 | 3.40E-01 |
| NMR-LIPO | XL-LDL-ApoB | - | 0.55[0.46,0.66] | 1.80E-10 | 0.62[0.5,0.77] | 1.80E-05 | -0.07 | 1.10E-06 |
| NMR-LIPO | XL-LDL-cholesterol | - | 0.58[0.48,0.69] | 1.80E-09 | 0.63[0.51,0.78] | 2.50E-05 | -0.07 | 3.00E-07 |
| NMR-LIPO | XL-LDL-free cholesterol | - | 0.59[0.49,0.7] | 6.20E-09 | 0.62[0.5,0.77] | 1.10E-05 | -0.08 | 3.50E-08 |
| NMR-LIPO | XL-LDL-phospholipids | - | 0.47[0.38,0.57] | 2.60E-13 | 0.62[0.49,0.78] | 5.00E-05 | -0.07 | 2.10E-06 |
| NMR-LIPO | L-LDL-ApoB | - | 0.61[0.52,0.72] | 3.40E-09 | 0.75[0.62,0.92] | 4.50E-03 | -0.04 | 7.50E-03 |
| NMR-LIPO | L-LDL-cholesterol | - | 0.53[0.45,0.63] | 8.10E-14 | 0.73[0.6,0.89] | 1.70E-03 | -0.05 | 1.90E-04 |
| NMR-LIPO | L-LDL-free cholesterol | - | 0.54[0.45,0.64] | 3.00E-12 | 0.68[0.56,0.84] | 2.40E-04 | -0.06 | 1.80E-05 |
| NMR-LIPO | L-LDL-phospholipids | - | 0.58[0.49,0.68] | 5.00E-11 | 0.73[0.61,0.89] | 1.70E-03 | -0.04 | 1.30E-03 |
| NMR-LIPO | L-LDL-triglycerides | - | 1.6[1.35,1.9] | 6.60E-08 | 1.17[0.96,1.43] | 1.10E-01 | 0.05 | 7.40E-04 |
| NMR-LIPO | M-LDL-triglycerides | - | 1.74[1.46,2.08] | 1.10E-09 | 1.47[1.2,1.81] | 2.40E-04 | 0.07 | 8.90E-08 |
| NMR-LIPO | S-LDL-ApoB | - | 1.61[1.38,1.87] | 4.90E-10 | 1.85[1.56,2.21] | 6.20E-12 | 0.11 | 3.80E-15 |
| NMR-LIPO | S-LDL-cholesterol | - | 1.57[1.34,1.83] | 2.00E-08 | 1.93[1.61,2.33] | 4.10E-12 | 0.11 | 5.10E-15 |
| NMR-LIPO | S-LDL-free cholesterol | - | 1.51[1.29,1.78] | 5.90E-07 | 1.85[1.53,2.24] | 3.20E-10 | 0.11 | 5.10E-14 |
| NMR-LIPO | S-LDL-phospholipids | - | 1.58[1.36,1.85] | 4.70E-09 | 1.89[1.57,2.27] | 9.30E-12 | 0.12 | 3.50E-16 |
| NMR-LIPO | S-LDL-triglycerides | - | 2.1[1.75,2.53] | 4.20E-15 | 1.73[1.39,2.15] | 7.90E-07 | 0.09 | 6.50E-12 |
| NMR-LIPO | XS-LDL-ApoB | - | 1.42[1.22,1.65] | 3.60E-06 | 1.56[1.31,1.85] | 3.70E-07 | 0.05 | 2.20E-04 |
| NMR-LIPO | XS-LDL-cholesterol | - | 1.35[1.16,1.57] | 1.00E-04 | 1.52[1.28,1.81] | 2.40E-06 | 0.06 | 4.70E-05 |
| NMR-LIPO | XS-LDL-phospholipids | - | 1.4[1.21,1.63] | 7.70E-06 | 1.56[1.31,1.86] | 3.80E-07 | 0.06 | 3.90E-06 |
| NMR-LIPO | XS-LDL-triglycerides | - | 1.61[1.39,1.86] | 2.10E-10 | 1.5[1.26,1.78] | 5.70E-06 | 0.07 | 7.10E-07 |
| NMR-LIPO | HDL-free cholesterol | - | 0.55[0.45,0.67] | 1.20E-09 | 0.64[0.51,0.8] | 1.10E-04 | -0.09 | 8.40E-10 |
| NMR-LIPO | HDL-triglycerides | - | 1.65[1.36,2.03] | 5.90E-07 | 1.3[1.04,1.62] | 2.20E-02 | 0.04 | 8.70E-03 |
| NMR-LIPO | L-HDL-ApoA2 | - | 1.38[1.16,1.64] | 0.00021 | 1.01[0.81,1.24] | 9.40E-01 | 0.02 | 1.40E-01 |
| NMR-LIPO | L-HDL-free cholesterol | - | 0.51[0.42,0.62] | 3.90E-12 | 0.66[0.53,0.81] | 1.50E-04 | -0.09 | 3.20E-10 |
| NMR-LIPO | M-HDL-ApoA2 | - | 1.4[1.19,1.65] | 5.80E-05 | 1.05[0.86,1.28] | 6.10E-01 | 0.04 | 4.10E-03 |
| NMR-LIPO | M-HDL-triglycerides | - | 1.44[1.21,1.7] | 3.10E-05 | 1.27[1.03,1.56] | 2.30E-02 | 0.04 | 7.00E-03 |
| NMR-LIPO | S-HDL-triglycerides | - | 1.52[1.29,1.77] | 2.20E-07 | 1.53[1.26,1.85] | 1.50E-05 | 0.06 | 1.80E-05 |

Odds ratio (OR) and 95% confidence interval (CI) estimates provided from logistic regression and Effect from linear regression with age- sex- and lipid-lowering medication-adjusted in the standardized metabolite variables. (P): positive association in previous study; (N): negative association in previous study.
