# Supplementary material for: Metabolomics based markers predict type 2 diabetes in a 14-year follow-up study
Source: Metabolomics. 2017 Jul 28;13(9):104. doi: 10.1007/s11306-017-1239-2 (PMC5533833; doi:10.1007/s11306-017-1239-2)
Supplement: Supplementary file 2 — Supplementary material 2 (DOCX 1760 KB) [file 11306_2017_1239_MOESM2_ESM.docx]

**Supplementary Figure 1** The specificity with fixed 80% sensitivity in different models and subgroups.

**
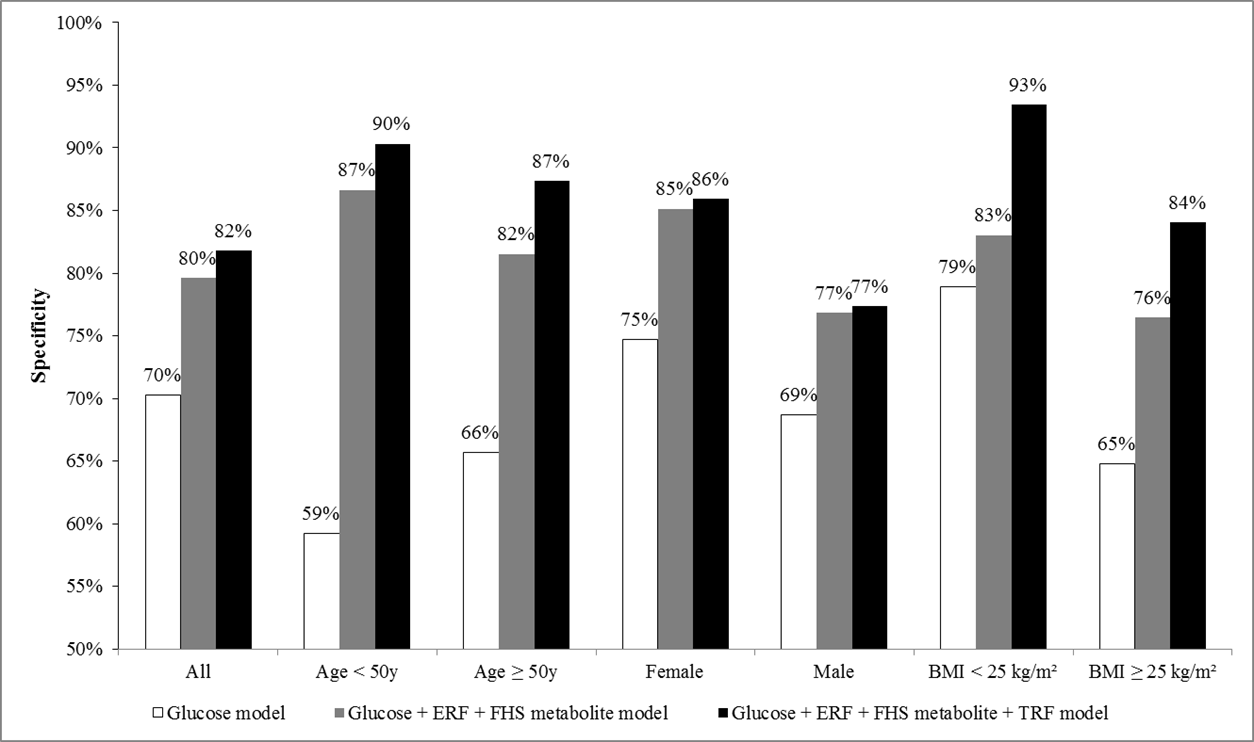
**

X axis includes different groups and models. Y axis is the specificity with the sensitivity of the models fixed to 80% in different groups.

**Supplementary Figure 2** ROC and AUC of different models in the follow-up dataset.


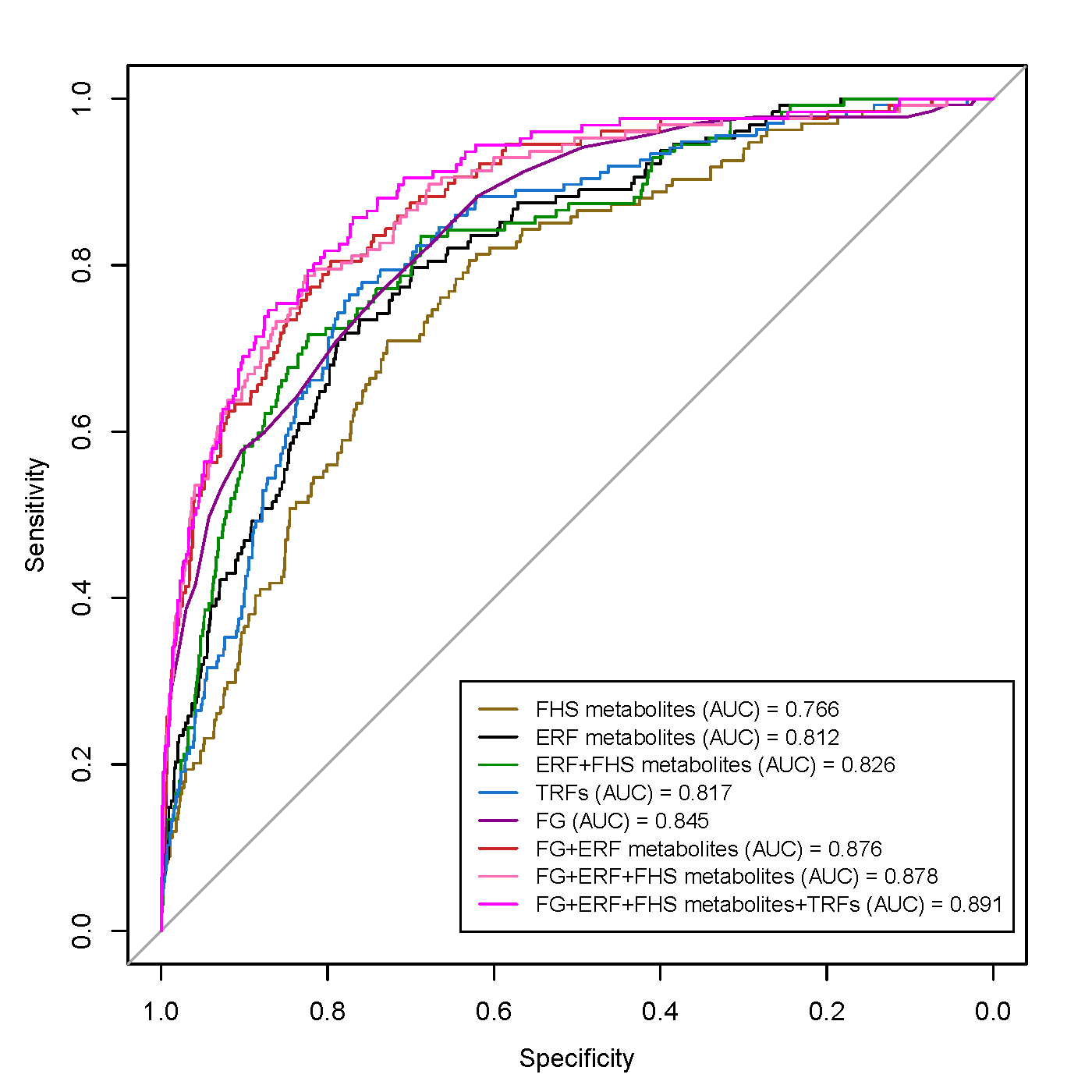


**Supplementary Figure 3** ROC and AUC of metabolite model and fasting glucose model in different subgroups.


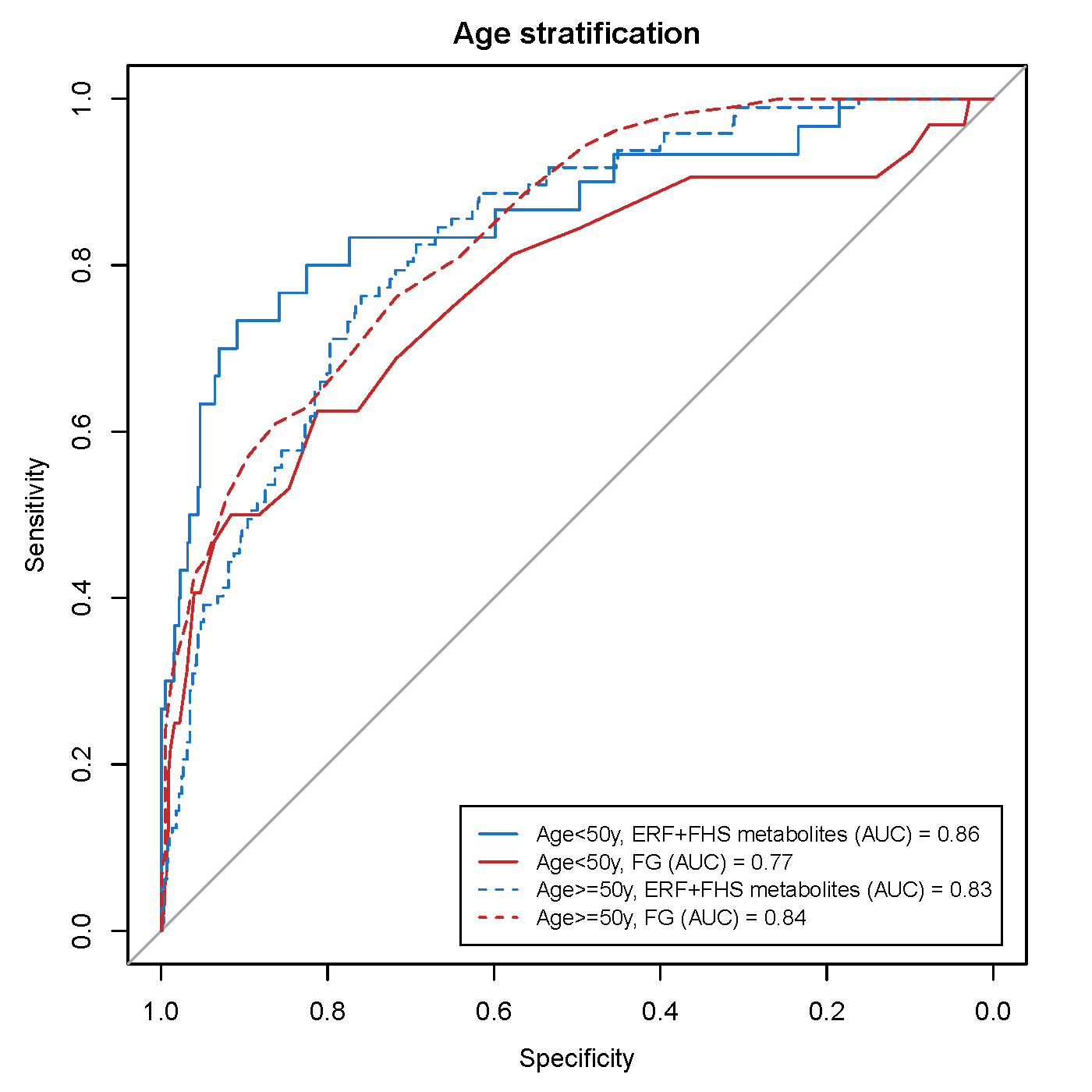


**
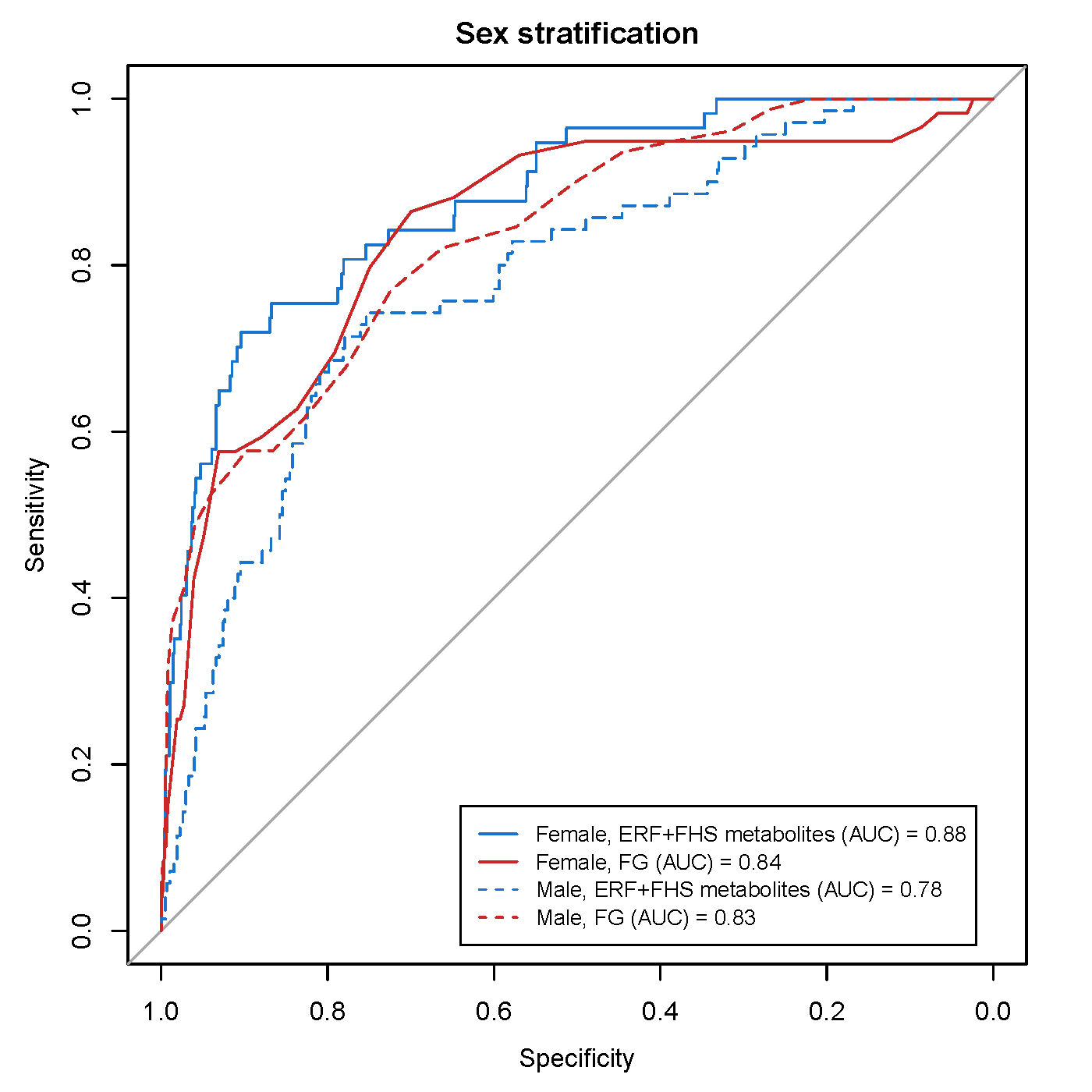
**

**
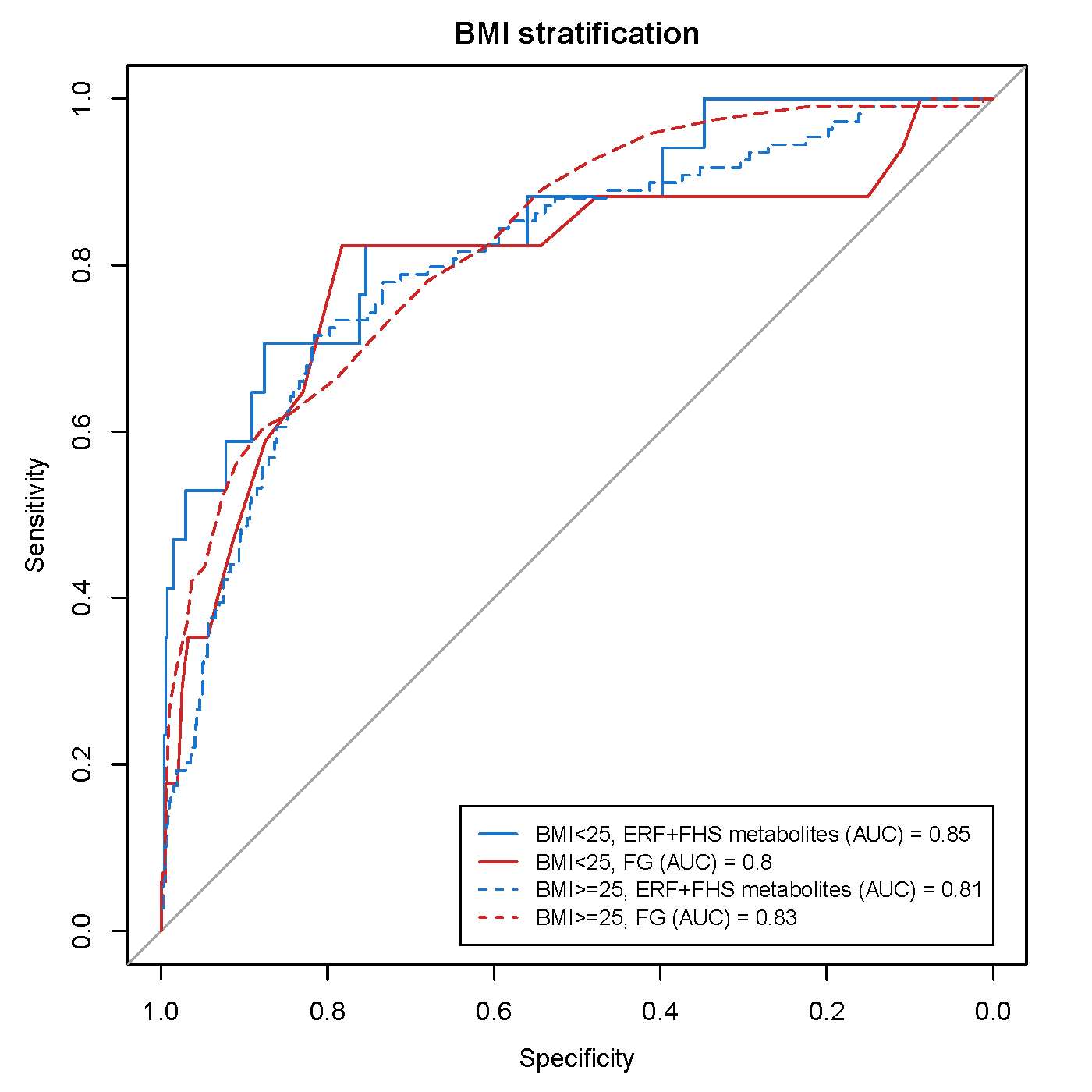
**

**Supplementary Figure 4** AUC of different models in different subgroups based on Cox model.


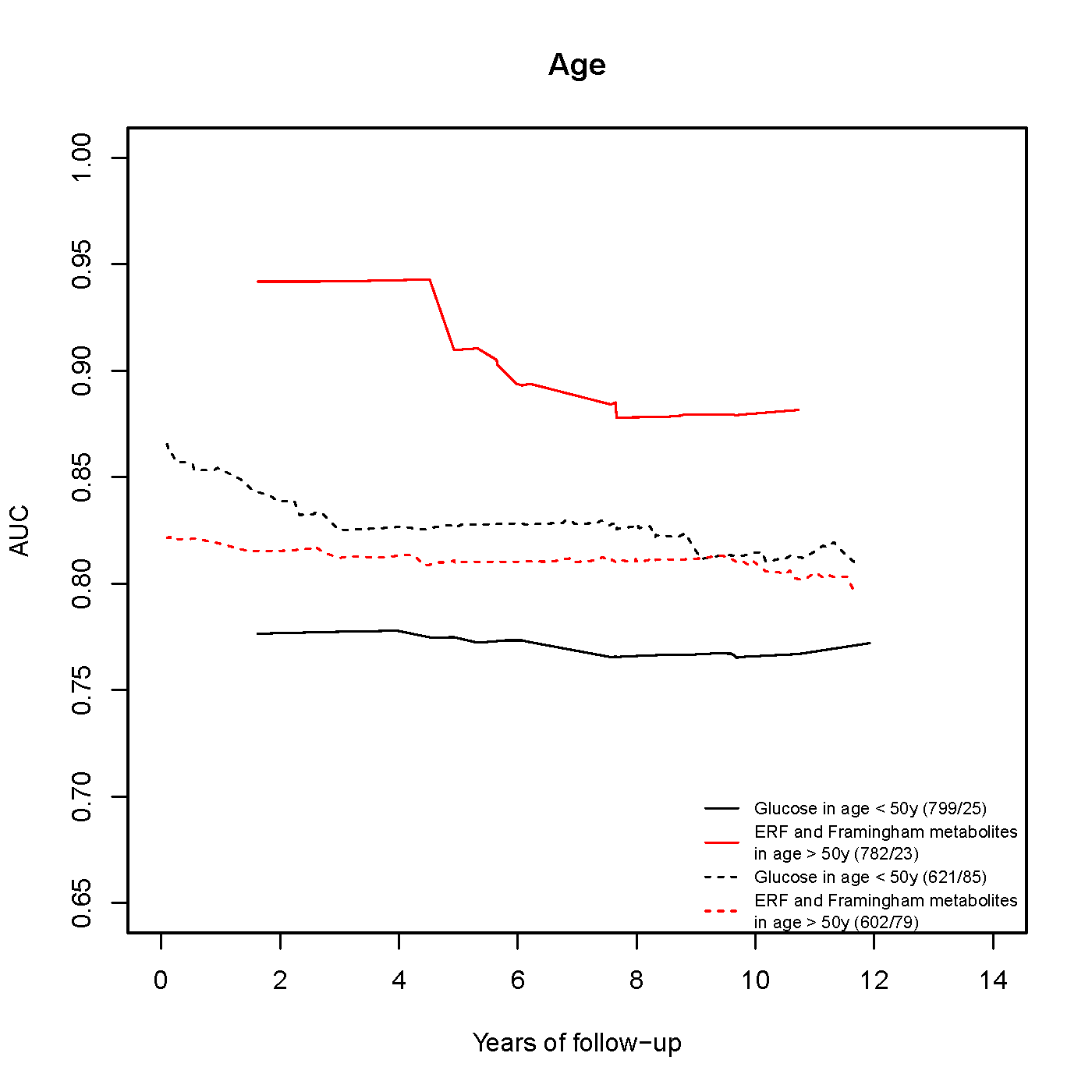


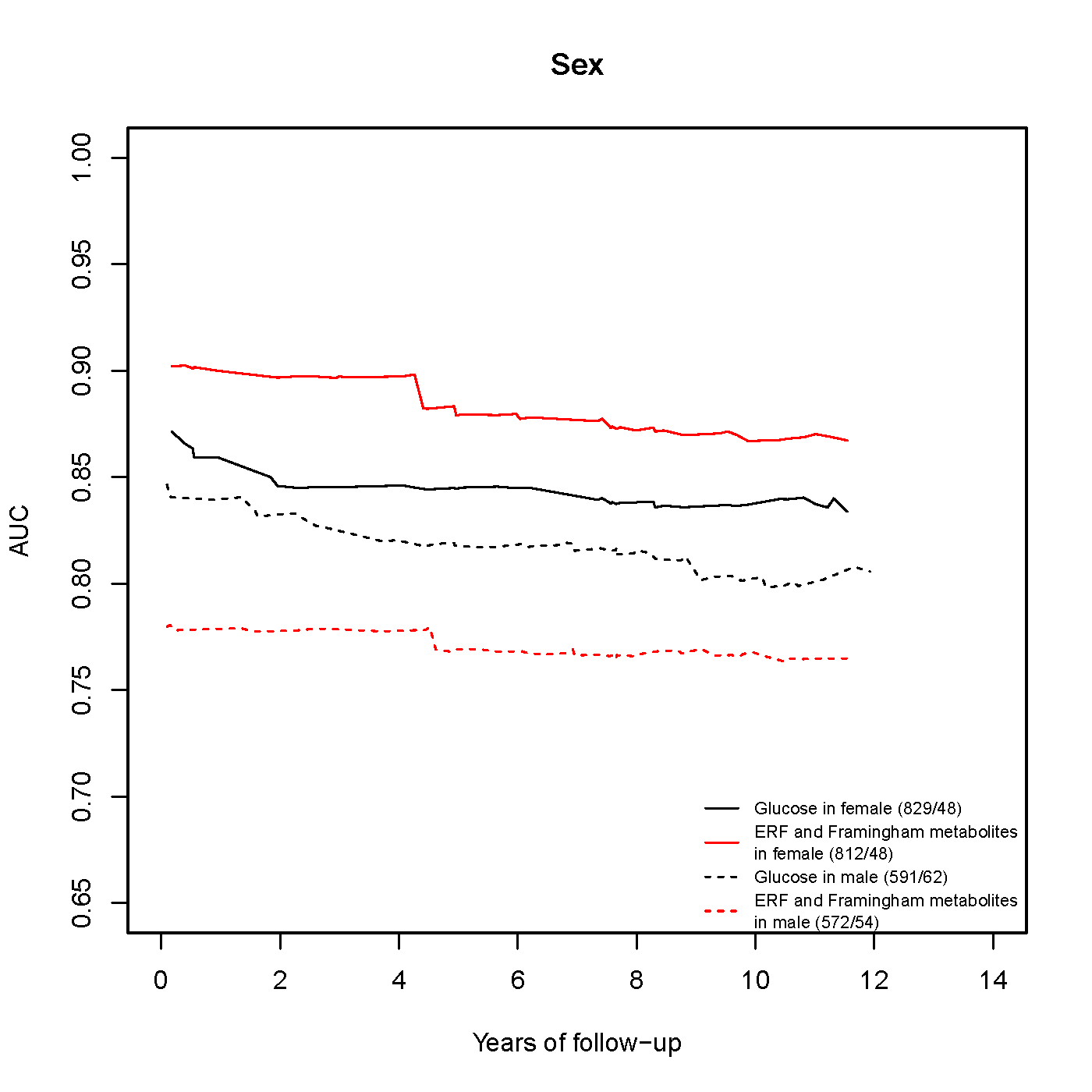


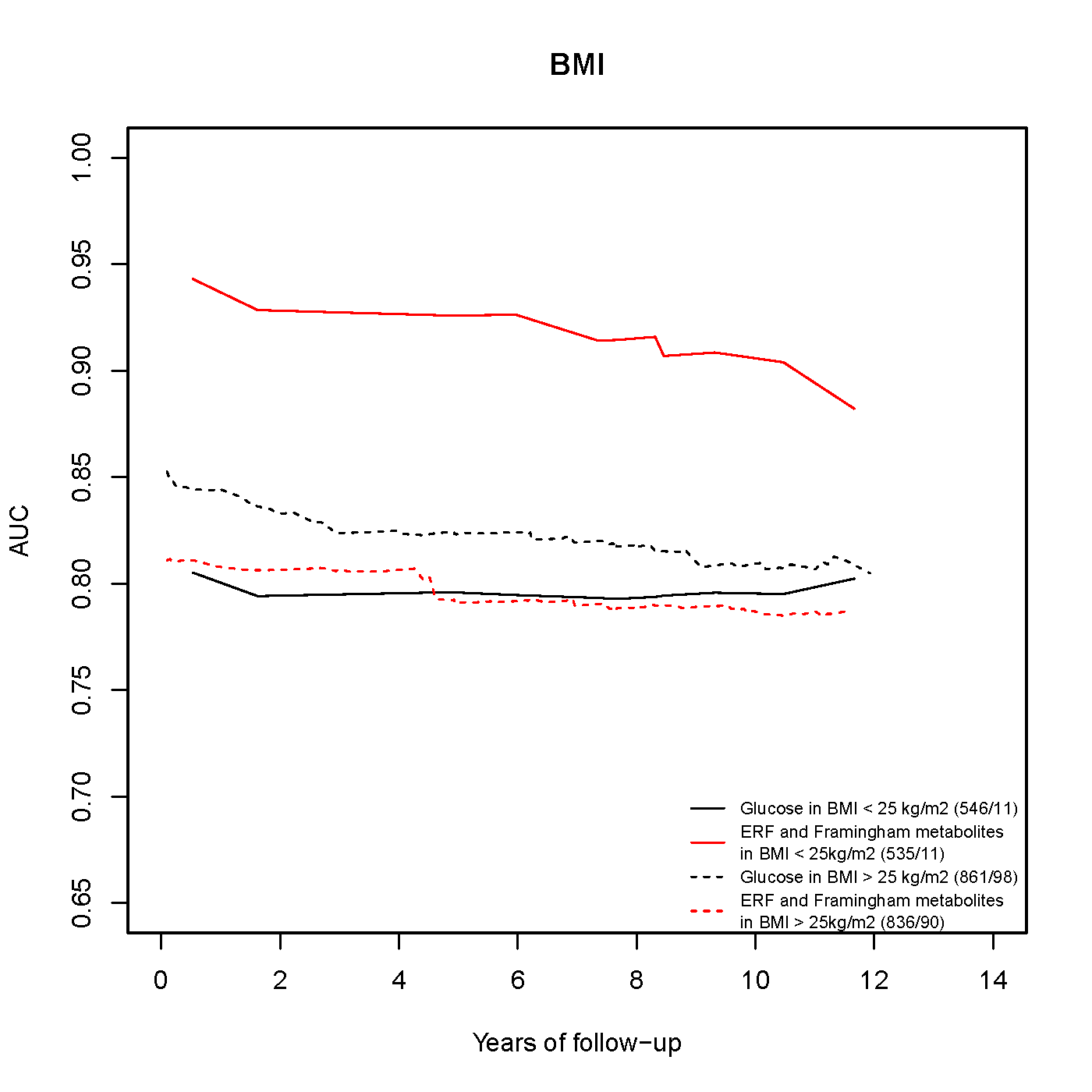


(/): Number of controls and incident cases analyzed in the follow-up.

The time of follow-up was derived from the time of the baseline examination to the onset of T2DM in the incident cases, and date of death, or the end of follow-up in the non-diabetes. The incident AUC based on Cox model was plotted with the function in *risksetROC* package in R in the follow-up individuals with complete information (n = 1,475, cases = 103). (1)

**Supplementary Figure 5** The consistency across Z scores of the metabolites measured in duplication between different platforms.

ESI-MS: Electrospray-Ionization MS. Biocrates: AbsoluteIDQTM p150 Kit of Biocrates Life Sciences AG. Sixty-two phospholipids are overlapping between LC-MS and ESI-MS. For all of the type 2 diabetes associated metabolites selected by LC-MS in the present study, the directions of effects were consistent across the two datasets. Nine amino acids were measured by both NMR-COMP and Biocrates. For all the type 2 diabetes associated metabolites selected by NMR-COMP, except methionine, all other metabolites have same direction of effect in both datasets.

**Reference**

1. Heagerty PJ, Zheng Y. Survival model predictive accuracy and ROC curves. Biometrics. 2005;61(1):92-105.
