# Supplementary material for: Metabolomics based markers predict type 2 diabetes in a 14-year follow-up study
Source: Metabolomics. 2017 Jul 28;13(9):104. doi: 10.1007/s11306-017-1239-2 (PMC5533833; doi:10.1007/s11306-017-1239-2)
Supplement: Supplementary file 3 — Supplementary material 3 (DOCX 758 KB) [file 11306_2017_1239_MOESM3_ESM.docx]

*Quality control (QC) of samples in different platforms*

*LC-MS measurements*

For the metabolites in LC-MS platform, relative ratios (RR) of each lipid species were obtained by dividing the area under the curve of each lipid by the area under the curve of its assigned internal standard. RR were corrected for intra- and inter-batch variation prior to statistical analyses and the details of this approach are given in the previous study (van der Kloet, Bobeldijk, Verheij, Jellema 2009). In brief, it is a workflow for significant reduction of the analytical error using pooled calibration samples and multiple internal standard strategy. Between and within batch calibration techniques are applied and the analytical error is reduced significantly (increase of 25% of peaks with relative standard deviations (RSD) lower than 20%) and does not hamper or interfere with statistical analysis of the final data. Figure 1 shows the QC for the example lipid PC (34:2). After correction, the QC samples tend to have stable RR. The correction plots for the remain lipids are available upon request.

*NMR–COMP measurements*

The NMR-COMP measurement used in the present study has been automatized and applied in more than 11,000 samples from six cohorts including the ERF population and the method is extensively described in a recent methodological paper (Verhoeven, Slagboom, Wuhrer, Giera, Mayboroda 2017). The performance of the method was assessed by comparing the calculated areas of the various glucose peaks with known clinical values (CVs), by comparing several peaks of the same metabolite (extracted versus non-extracted), and by comparing areas obtained from various NMR pulse sequences and spiking experiments. Meanwhile, 174 QC samples were randomly distributed during the measurements and were subsequently processed in exactly the same way as the samples of the actual study, and hence, no additional normalization was performed with QCs. The calculated CVs (%) of the representative metabolic measures as shown below exhibit a mean CV of 7.04 % (+/- 3.85) and a median CV of 5.92 %, with only 4 measures having a CV more than 10 %. This outcome clearly suggests the lack of any trend within the 174 QC samples and good reproducibility of the QC samples across the study. Table 1 depicts the most representative chemical shift values for the metabolic measures in the NMR spectra (many metabolites have a complicated signal with components at multiple chemical shift locations).

*NMR–LIPO measurements*

These measurements have been performed by Bruker Ltd. using a standard commercial algorithm of which the details have been kept confidential. The overall method is described in: [https://www.bruker.com/fileadmin/user_upload/8-PDF-Docs/MagneticResonance/NMR/brochures/lipo-analysis_apps.pdf](https://www.bruker.com/fileadmin/user_upload/8-PDF-Docs/MagneticResonance/NMR/brochures/lipo-analysis_apps.pdf)). However, being the end user of this data, we were not given access to details on QC procedures from Bruker.

**Figure 1** The QC of PC (34:2) in LC-MS.


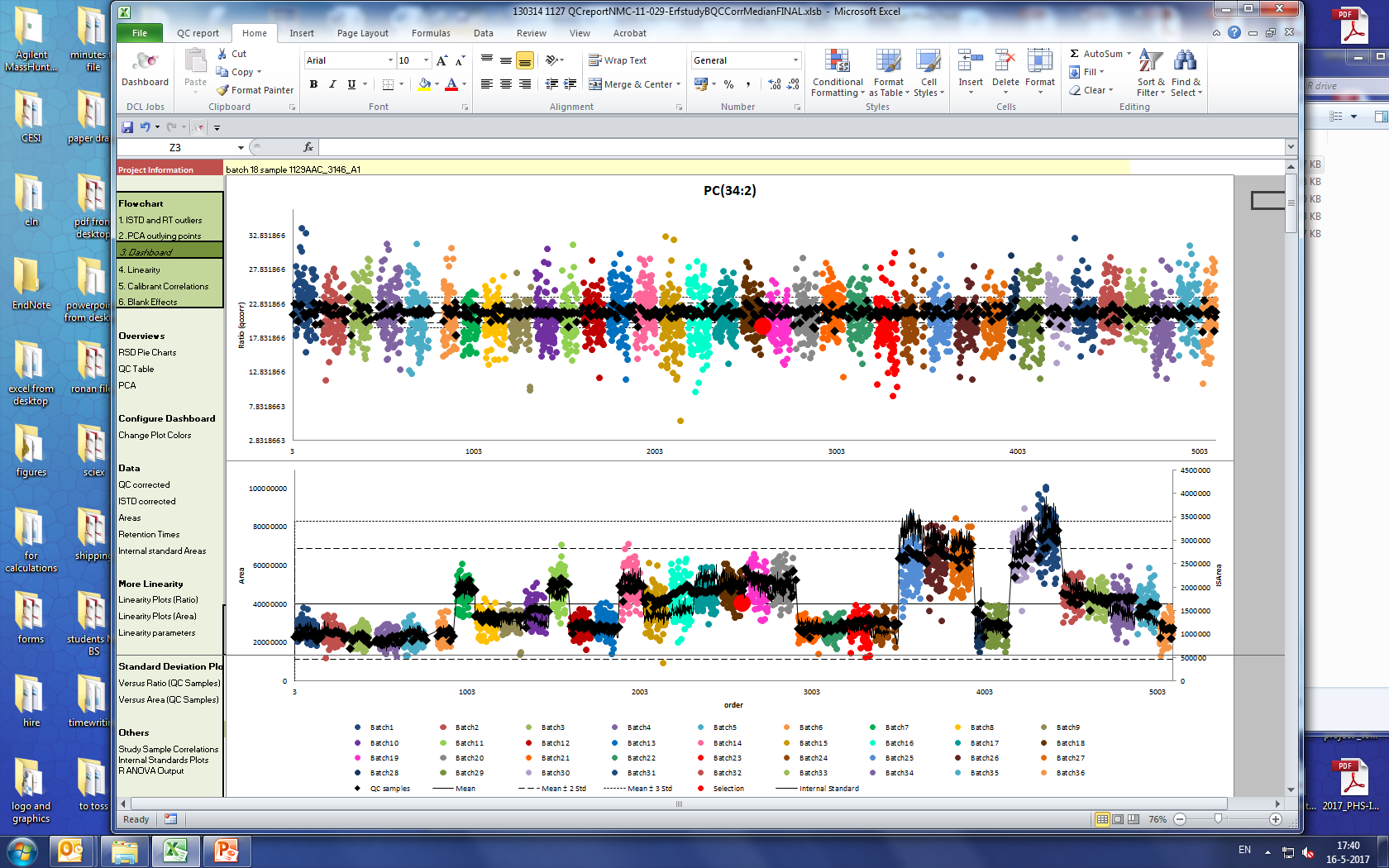


The colored dots are the study samples; the black dots are the QC samples. The top plot shows the values after correction, whereas the bottom plot shows uncorrected peak areas.

**Table 1** The most representative chemical shift values (CS) for the metabolic measures in the NMR-COMP.

| Measure | CS / ppm | CV / % |
| --- | --- | --- |
| Leucine | 0.95 | 3.91 |
| Isoleucine | 1.00 | 4.45 |
| Valine | 1.03 | 3.96 |
| 3-Hydroxybutyrate | 1.19 | 6.49 |
| Lipids (CH2) | 1.27 | 6.17 |
| Alanine | 1.47 | 4.38 |
| Acetate | 1.91 | 8.26 |
| Pyruvate / Oxaloactetate | 2.36 | 5.52 |
| Glutamine | 2.47 | 11.0 |
| Citrate | 2.53 | 5.88 |
| Glycine | 3.55 | 5.97 |
| Glycerol | 3.56 | 18.67 |
| Creatinine | 4.05 | 5.08 |
| Lactate | 4.10 | 4.68 |
| Lipids (CH=CH*CH2HC2) | 5.30 | 12.01 |
| Tyrosine | 6.89 | 6.41 |
| Phenylalanine | 7.42 | 10.53 |

**References**

van der Kloet, F. M., I. Bobeldijk, E. R. Verheij, R. H. Jellema (2009). Analytical error reduction using single point calibration for accurate and precise metabolomic phenotyping. J Proteome Res 8, 5132-41

Verhoeven, A., E. Slagboom, M. Wuhrer, M. Giera, O. A. Mayboroda (2017). Automated quantification of metabolites in blood-derived samples by NMR. Analytica Chimica Acta,
